# Poge heart-saving decoction meliorates heart failure by suppressing apoptosis and fibrosis via regulation of the PI3K/AKT pathway

**DOI:** 10.3389/fphar.2026.1748420

**Published:** 2026-03-25

**Authors:** Lei Du, Luquan Qin, Ailing Zhou, Yi Ren, Shumei Wang

**Affiliations:** 1 College of Traditional Chinese Medicine, Chongqing Medical University, Chongqing, China; 2 Classics of Traditional Chinese Medicine, Chongqing Hospital of Traditional Chinese Medicine, Chongqing, China

**Keywords:** heart failure, myocardial apoptosis, myocardial fibrosis, PI3K/Akt pathway, poge heart-saving decoction

## Abstract

**Background:**

Poge Heart-Saving Decoction (PHSD) is a traditional Chinese medicine formulation that has been used clinically for decades in the treatment of heart failure (HF). However, its precise therapeutic mechanisms remain incompletely understood.

**Methods:**

The metabolites of PHSD were characterized using UHPLC-MS/MS. Network analysis was subsequently employed to identify the mechanism. A mouse model of HF was established through intraperitoneal injection of isoproterenol (ISO), followed by treatment with PHSD at low, medium or high doses. Cardiac function parameters were evaluated by echocardiography, and NT-proBNP levels were measured. Histopathological examination of myocardial tissue was conducted, complemented by analyses of protein and mRNA expression levels related to apoptosis and fibrosis targeting the PI3K/AKT pathway.

**Results:**

A total of 133 metabolites in PHSD were identified. Network analysis suggested that PHSD may ameliorate HF by targeting key proteins such as AKT1, TNF, and BCL-2. *In vivo* experiments demonstrated that PHSD alleviated ISO-induced myocardial apoptosis by balanced BAX and BCL-2. Furthermore, PHSD significantly reduced the deposition of Collagen I and Collagen III and markedly downregulated the expression of PI3K and AKT.

**Conclusion:**

Our study demonstrated that PHSD ameliorates HF by suppressing myocardial apoptosis and fibrosis through inhibition of the PI3K/AKT pathway. These findings indicate that PHSD is a prospective therapeutic agent against HF.

## Graphical Abstract

**Figure:**
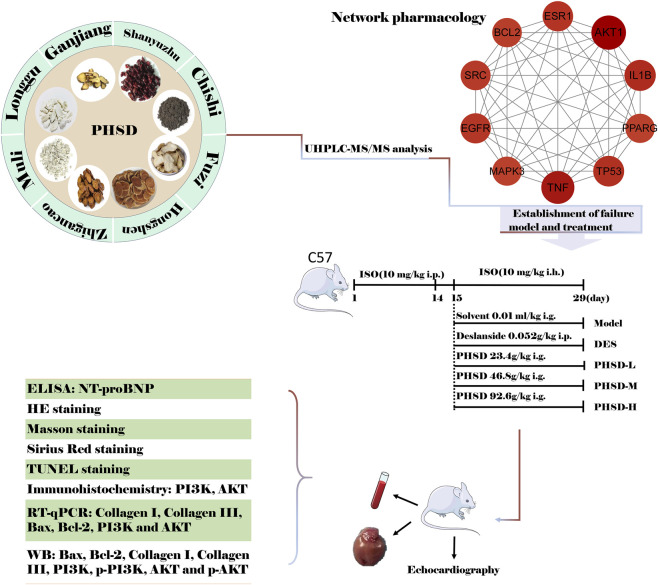

## 1 Introduction

Heart failure (HF) represents a clinical syndrome resulting from diverse pathological conditions that impair cardiac structure and/or function. Although significant therapeutic advancements have been made in HF management, both the incidence and mortality rates of HF remain persistently high (Chioncel et al., 2017; James et al., 2018; Wang et al., 2021). Cardiac remodeling is widely recognized as the fundamental pathological basis of HF (Cohn et al., 2000). Myocardial apoptosis and fibrosis play critical roles in cardiac remodeling (Zhang et al., 2022), and these processes are regulated by the phosphoinositide 3-kinase/protein kinase B (PI3K/AKT) pathway (Cui et al., 2024).

Cardiomyocyte apoptosis accelerates the progression of HF. Activation of the PI3K/AKT pathway induces cardiomyocyte apoptosis (Wang et al., 2023). Bax, a pivotal regulator of apoptosis, actively promotes programmed cell death (Dadsena et al., 2021). Current evidence demonstrates that Bax activation represents an essential step in mitochondrial outer membrane permeabilization (MOMP), a process that irreversibly commits cells to apoptosis (Jin et al., 2008; Spitz and Gavathiotis, 2022). Conversely, Bcl-2 serves as an endogenous apoptosis inhibitor (Singh et al., 2019), with studies confirming its substantial protection against myocardial injury and significantly improves left ventricular dysfunction following ischemia event (Chen et al., 2001; Chatterjee et al., 2002).

Myocardial fibrosis represents a pathological basis of cardiac remodeling (Qin W. et al., 2021). The PI3K/AKT signaling pathway has dual regulatory effects on myocardial cells (Packer, 2023). Under conditions of acute cardiac workload increases, activation of PI3K/AKT pathway serves to prevent excessive autophagy and autophagosome accumulation in cardiomyocytes, while concurrently stimulating mitochondrial synthesis and enhancing the antioxidant capacity to protect cardiomyocytes (Datta Chaudhuri et al., 2021; Qin G.-W. et al., 2021; Han et al., 2022). Conversely, persistent chronic cardiac overload results in chronic PI3K/AKT pathway activation, which subsequently promotes sustained inflammatory responses and the development of myocardial fibrosis (Wende et al., 2015; Sciarretta et al., 2022). These pathophysiological alterations are mechanistically linked to cardiac fibroblast activation and pathological extracellular matrix (ECM) deposition (Qin W. et al., 2021).

Increasing evidence indicates that traditional Chinese medicine (TCM) plays a significant role in alleviating HF. Poge Heart-Saving Decoction (PHSD), developed by a distinguished TCM-expert professor Ke Li, consists of eight herbs: Fuzi (Aconite Tuber), Ganjiang (Ginger Rhizome), Hongshen (Red Ginseng), Shanzhuyu (Cornus), Gancao (Liquorice Root), Longgu (Ossa Draconis), Muli (Oyster), and Cishi (Magnetitum). Clinical studies have demonstrated PHSD’s significant therapeutic efficacy in HF treatment (Zhan et al., 2023; Zhou et al., 2024). Previous evidence suggests that PHSD may ameliorate HF through inhibition of the renin-angiotensin-aldosterone system (RAAS), consequently reducing aldosterone-mediated cardiac load (Liu et al., 2020). Nevertheless, the exact mechanisms responsible for its cardioprotective effects remain to be further elucidation.

In this study, a comprehensive methodological approach combining network analysis with *in vivo* experiments was employed, aiming to investigate the therapeutic effects and mechanisms of PHSD in HF.

## 2 Materials and methods

### 2.1 Herb materials

The eight herbs used in this study—Fuzi (Aconite Tuber), Ganjiang (Ginger Rhizome), Hongshen (Red Ginseng), Shanzhuyu (Cornus), Gancao (Liquorice Root), Longgu (Ossa Draconis), Muli (Oyster), and Cishi (Magnetitum)—were obtained as herbal concentrate granules from Sichuan New Green Pharmaceutical Technology Development Co., Ltd. (Sichuan, China). Detailed information on the herbs is presented in Table 1. The standard of each herbal granule is provided in Supplementary File 1.

**TABLE 1:** The herbs information of PHSD.

Chinese name	English name	Latin name	Family	Part	Dosage (g)	Origin and batch number
Fuzi	Aconite tuber	*Aconitum carmichaelii* Debeaux	Ranunculaceae	Root	60	Sichuan, China 21070204
Ganjiang	Ginger rhizome	*Zingiber officinale* Roscoe	Zingiberaceae	Dried rhizome	60	Sichuan, China 21080214
Hongshen	Red ginseng	*Panax ginseng* C.A.Me.	Araliaceae	Root and rhizome	60	Jilin, China 21100366
Shanzhuyu	Cornus	*Cornus officinalis* Siebold & Zucc.	Cornaceae	Sarcocarp	60	Henan, China 21100304
Gancao	Licorice	*Glycyrrhiza glabra* L.	Fabaceae	Root and rhizome	30	NeiMongol, China 21100285
Muli	Oyster shell	*Ostreae rivularis G*ould	Ostreidae	Shell	30	Liaoning, China 21080135
Cishi	Magnetite^a^	—	—	Whole part	30	Shandong, China 21100197
Longgu^b^	Fossilia Ossia Mastodi	—	—	Fossil	30	Henan, China 21090013

^a^ Magnetite is a member of the spinel group of oxide minerals, primarily composed of Fe_3_O_4_.

^b^ Longgu refers to fossilized bone originating from large mammals, such as mammoths.

### 2.2 Ultra-high-performance liquid chromatography-tandem mass spectrometry (UHPLC-MS/MS) analysis

The sample was precisely weighed and mixed with 1 mL of 50% methanol aqueous solution, followed by sonication for 5 min. The mixture was subsequently centrifuged at 22,000 × g for 5 min. The supernatant was filtered through a 0.22 μm microporous membrane and transferred to a sample vial for UHPLC-MS/MS analysis (U3000, Thermo Fisher, Massachusetts, United States). Chromatographic separation was achieved using an ACQUITY UPLC HSS T3 column (2.1 × 100 mm, 1.8 μm) maintained at 35 °C. The injection volume was 10 μL, with a mobile phase flow rate of 0.3 mL/min. The gradient elution conditions are specified in Supplementary File 2, Table B1. Mass spectrometric detection was performed using a Q Exactive Plus Orbitrap mass spectrometer (Thermo Fisher, Massachusetts, United States) operating in Full MS-ddMS2 mode, with data acquisition in both positive and negative ion modes. The raw mass spectrometry data were processed using Compound Discoverer 3.2 software.

### 2.3 Network analysis

Metabolites identified by UHPLC–MS/MS were screened according to oral bioavailability (OB) > 30% and drug-likeness (DL) > 0.18 using the TCMSP database (https://www.tcmsp-e.com/tcmsp.php). Potential targets of screened metabolites were predicted via SwissTargetPrediction (https://swisstargetprediction.ch/). Heart failure-related targets were retrieved from the GeneCards database (https://www.genecards.org/) by searching the term “heart failure” with *Homo sapiens* specified as the species. The top 2,000 targets ranked by relevance score were selected for subsequent analysis. Overlapping targets between PHSD metabolites and HF were identified and visualized using E-Venn (https://www.bic.ac.cn/test/venn/#/). A protein–protein interaction (PPI) network of the shared targets was constructed through the STRING database (https://cn.string-db.org/) with a confidence score threshold of 0.4, excluding disconnected nodes. Core targets were determined based on the median values of degree, betweenness centrality, and closeness centrality, and the resulting network was visualized in Cytoscape 3.10.4. Gene Ontology (GO) and Kyoto Encyclopedia of Genes and Genomes (KEGG) enrichment analyses were performed using Metascape (Zhou et al., 2019), and the top 10 GO terms and top 20 KEGG pathways were visualized via Bioinformatics (https://www.bioinformatics.com.cn/).

### 2.4 Experimental validation

#### 2.4.1 Antibodies and reagents

Antibodies against PI3K (Cat.No. AF6242), p-PI3K (Cat.No. AF3241), Collagen I (Cat.No. AF7001), and Collagen III (Cat.No. AF5457) were obtained from Affinity (Jiangsu, China), while Bax (Cat.No. ET1603-34) and Bcl-2(Cat.No. ET1702-53) antibodies were acquired from Huabio (Hangzhou, China). Antibodies against AKT (Cat.No. 4691) and p-AKT (Cat.No. 4060) were purchased from Cell Signaling Technology (Massachusetts, United States). The NT-proBNP enzyme-linked immunosorbent assay (ELISA) kit was produced from MLbio (Cat.No. ml324452, Shanghai, China), with primers supplied by Western Biotechnology (Chongqing, China). Isoproterenol (Cat.No. HY-B0468) and deslanoside (Cat.No. HY-A0154) were purchased from MedChemExpress (New Jersey, United States). Additional specifications are provided in Supplementary File 2, Table B2.

#### 2.4.2 Animals

Six-week-old C57BL/6 mice (male, 20 ± 2 g) were procured from the Experimental Animal Center of Chongqing Medical University (Chongqing, China) and maintained under controlled conditions (12-h light/dark cycle, 23 °C ± 3 °C) with free access to standard chow (Lab mice diet, Cat. No. 1010001, Xietong Biotechnology Co., Ltd. Jiangsu, China) and water. All animal experimental procedures were performed in compliance with the Institutional Animal Care and Use Guidelines established by the Experimental Animal Center of Chongqing Medical University (No. IACUC-CQMU-2024-0330). Mice were housed in an individual cage system, with five mice per cage. Each cage was labeled with an identification tag, and mice were distinguished by marking their tails with a marker pen.

#### 2.4.3 Establishment of HF model and treatment

**FIGURE 1:**
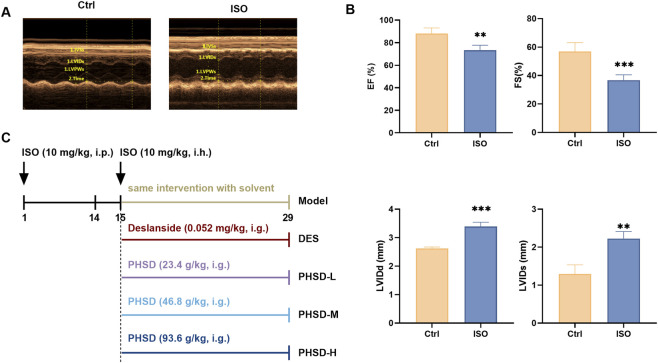
Model evaluation and experimental procedure diagram. **(A)** Representative echocardiography images of the control group and the ISO group. **(B)** Cardiac function parameters in the control group and ISO group (n = 5). **(C)** Experimental procedure diagram.

Sixty mice were randomly allocated into two groups: the isoproterenol (ISO) group (n = 50) and the normal control (NC) group (n = 10). The ISO group received isoproterenol (ISO, 10 mg/kg, intraperitoneal injection) (Sun et al., 2023) to establish the HF model, while the NC group was administered an equal volume of saline for 14 consecutive days. Cardiac function were measured by echocardiography (Sun et al., 2023). Echocardiography showed that LVIDs and LVIDd were increased, accompanied by decreased EF and FS values in the model group (P < 0.05, Figures 1A,B), suggesting the development of HF. After successful model establishment, the HF model mice were randomly divided into five groups: model group, positive (deslanoside, DES) group, and low, medium, and high dose PHSD (PHSD-L, PHSD-M, and PHSD-H) groups (n = 10 per group). Deslanoside is a widely used drug for treat HF. It exerts a marked positive inotropic effect by enhancing myocardial contractility, increasing cardiac output, and slowing the heart rate, thereby improving cardiac function (Miller et al., 2002). All animals in the five groups continued to receive ISO (10 mg/kg, i.h.) for an additional 14 days. During this period, the DES group was treated with deslanoside (0.052 mg/kg, i.p.). The PHSD-L, PHSD-M, and PHSD-H groups were treated with PHSD at 0.5 × (23.4 g/kg), 1 × (46.8 g/kg), and 2 × (93.6 g/kg, i.g.) the equivalent clinical dose crude herbal material (Reagan-Shaw et al., 2008). The detailed dose calculation is provided in Supplementary File 2, Table B3. A schematic diagram of the *in vivo* experimental design is presented in Figure 1C.

#### 2.4.4 Echocardiography

After the last administration, cardiac function was assessed using a research-specific color Doppler ultrasound diagnostic device (VINNO, D6 LAB, Jiangsu, China). The following parameters were recorded: left ventricular internal diameter at end-diastole (LVIDd), left ventricular internal diameter at end-systole (LVIDs), ejection fraction (EF) and fractional shortening (FS).

#### 2.4.5 ELISA assay

Following echocardiography, mice were anesthetized with sodium pentobarbital (40 mg/kg, i.p.), and blood samples were obtained by enucleation of the eyeball. The animals were subsequently euthanized by cervical dislocation, and their hearts were harvested for downstream analyses. Serum NT-proBNP levels were measured using a mouse NT-proBNP assay kit (ml324452, MLbio, Shanghai, China) according to the instructions. Blood samples were centrifuged at 3,000 × g for 10 min, and the supernatant was collected. Standard wells were loaded with 50 µL of standard solution at varying concentrations. For sample wells, 10 µL of sample was added, followed by 40 µL of sample dilution buffer. Subsequently, 100 µL of enzyme-conjugated reagent was added to each well. The plate was sealed with adhesive film and incubated at 37 °C for 1 h. After incubation, the liquid was discarded, and the plate was patted dry. The plate was washed 5 times for 1 min each with wash buffer. Next, 50 µL each of substrate A and substrate B were added to all wells, followed by 15 min of incubation in the dark at 37 °C. The reaction was stopped with 50 µL of stop solution. Optical density (OD) was measured at 450 nm.

#### 2.4.6 Hematoxylin-eosin (HE) staining

HE staining was performed according to the instructions of the Hematoxylin and Eosin staining kit (Cat.No. C0105S, Beyotime, Shanghai, China). The cardiac tissues were rinsed with PBS to remove residual blood and fixed in 4% paraformaldehyde. Fixed tissues were sequentially dehydrated through graded ethanol solutions (75%, 85%, 95% I, 95% II, 100% I, and 100% II) for 10 min each. Dehydrated tissues were cleared by immersion in xylene I and II for 10 min each. Following clearing, tissues were embedded in molten paraffin at 60 °C for 3 h. The resulting tissue blocks were sectioned at 5 μm thickness using a microtome (YD-315, Jinhua YIDI Medical Appliance Co., Ltd., Zhejiang, China), mounted onto adhesive slides, and then dried at 55 °C. For deparaffinization, sections were sequentially immersed in xylene I and xylene II for 10 min each. Rehydration was achieved through graded ethanol solutions (100% I, 100% II, 95% I, 95% II, 85%, and 75% for 5 min each). Slides were washed three times with distilled water (2 min each). Histological staining commenced with hematoxylin (10 min), followed by rinsing with tap water (5 min) and brief distilled water washing. Cytoplasmic overstaining was removed using 1% acid-alcohol. Sections were counterstained with eosin (30 s), with excess stain removed by tap water washing (3 min), and final distilled water rinsing. Processed slides were sealed with neutral resin for microscope examination (DMC5400, Leica, Wetzlar, Germany), revealing characteristic blue nuclei and pink cytoplasm.

#### 2.4.7 Masson staining

The preparation of sections prior to staining followed the same procedure as described in the HE staining section. Sections were stained with Weigert’s iron hematoxylin for 5 min, rinsed in phosphomolybdic acid solution for 2 min, and subsequently stained with aniline blue for 2 min. At each processing step, sections were washed with weak acid solution for 1 min. Following staining, sections were dehydrated through an ethanol series, cleared in xylene, and sealed with neutral resin. Microscopic examination and photography were then performed for further analysis.

#### 2.4.8 Sirius Red staining

The preparation of sections prior to staining followed the same procedure as HE staining. Sections were stained with Sirius Red solution for 1 h, then rinsed with running water to remove excess stain from the slide surface. The sections were subsequently counterstained with Mayer’s hematoxylin solution for 8 min to visualize cell nuclei. Following a 5 min rinse under running water, slides were sealed with neutral resin and examined microscopically for observation and photography.

#### 2.4.9 TUNEL staining

The sections were incubated with proteinase K at 37 °C for 30 min, followed by treatment with 3% hydrogen peroxide solution at room temperature for 20 min. Subsequently, a biotinylated reagent was applied to the sections, which were then incubated at 37 °C for 1 h while protected from light. After terminating the reaction, streptavidin-HRP solution was added and incubated at room temperature for 30 min. DAB chromogenic solution was applied and incubated at room temperature for 15 min. The sections were counterstained with hematoxylin to highlight the cell nuclei. After sealing, the slides were examined and imaged under a microscope.

#### 2.4.10 Immunohistochemistry

Following dewaxing, sections were immersed in citrate buffer and subjected to microwave heating while maintaining the buffer temperature at 96 °C for 15 min to achieve optimal antigen retrieval. Slides were washed three times with PBS. Tissue permeabilization was performed by incubating sections with 0.5% Triton X-100 for 20 min at room temperature, followed by PBS washes. Endogenous peroxidase activity was blocked through incubation with 3% hydrogen peroxide (H_2_O_2_), followed by three PBS washes. Primary antibodies (PI3K diluted 1:150; AKT diluted 1:300) were applied to sections and incubated either overnight at 4 °C. After thorough washing, the sections were then incubated with HRP-conjugated secondary antibody (1:50 dilution) for 1 h at room temperature. Sections were incubated with DAB chromogenic solution under light-protected conditions. After chromogenic development, sections were rinsed twice with distilled water and counterstained with hematoxylin for 5 min. Slides were rinsed under running tap water to achieve tissue bluing, dehydrated through a graded ethanol series, cleared in xylene, and sealed with neutral resin. Finally, sections were examined by a light microscope and photographed for subsequent analysis (Al-Botaty et al., 2022).

#### 2.4.11 RT-qPCR

Approximately 30 mg of myocardial tissue was used for RNA extraction. Total RNA was extracted following the instructions of RNA extraction kit (9109, Takara, Japan). RNA concentration was measured with a NanoDrop spectrophotometer (NanoDrop Eight, Thermo Fisher, Massachusetts, United States). Complementary DNA (cDNA) was synthesized following the instructions of a commercial kit (11139ES10, Yeasen, Shanghai, China). Primer sequences are provided in Table 2. Quantitative PCR was performed on an Applied Biosystems real-time PCR system (Thermo Fisher, Massachusetts, United States) to quantify mRNA expression levels of Collagen I, Collagen III, Bax, Bcl-2, Pi3k and Akt, with the qPCR master mixture (11184ES03, Yeasen, Shanghai, China).

**TABLE 2:** Primer information.

Gene	GenBank accession	Primer sequence	Length (bp)
M-*Gapdh*-F	NM_001411845.1	5′-GGT​GAA​GGT​CGG​TGT​GAA​CG-3′	233
M-*Gapdh*-R		5′-CTC​GCT​CCT​GGA​AGA​TGG​TG-3′	
M-*Bax*-F	NM_007527.4	5′-GGA​CGA​ACT​GGA​CAG​TAA​CAT​GG-3′	150
M-*Bax*-R		5′-GCA​AAG​TAG​AAA​AGG​GCG​ACA​AC-3′	
M-*Bcl 2*-F	NM_177410.3	5′-AAC​ATC​GCC​CTG​TGG​ATG​AC-3′	191
M-*Bcl 2*-R		5′-TAT​GCA​CCC​AGA​GTG​ATG​CAG-3′	
M-*Collagen I*-F	BC050014.1	5′-AGC​ACG​TCT​GGT​TTG​GAG​AG-3′	112
M-*Collagen I*-R		5′-GAC​ATT​AGG​CGC​AGG​AAG​GT-3′	
M-*Collagen III*-F	NM_009930.2	5′-AAA​GAG​GAT​CTG​AGG​GCT​CG-3′	141
M-*Collagen III*-R		5′-AGG​GTG​AAA​AGC​CAC​CAG​AC-3′	
M-*Pi3k*-F	NM_001077495.2	5′-GAG​TGC​AGA​GGG​CTA​CCA​GT-3′	92
M-*Pi3k*-R		5′-CAG​TCA​GTA​TGT​CCC​CCA​GG-3′	
M-*Akt*-F	NM_001331107.2	5′-AAG​GAG​ATC​ATG​CAG​CAC​CG-3′	195
M-*Akt*-R		5′-CTC​ACT​GTC​CAC​ACA​CTC​CA-3′	

#### 2.4.12 Western blotting (WB) assay

Myocardial tissue was homogenized in RIPA lysis buffer on ice using a glass homogenizer, followed by centrifugation at 16,099 × g for 5 min at 4 °C. Protein concentration was determined using a BCA protein assay kit (T9300A, Takara, Kyoto, Japan). SDS-PAGE electrophoresis was initially performed at 80 V for 20 min, and then at 120 V for 1 h. Proteins were subsequently transferred from the gel to a PVDF membrane at 200 mA for 1–3 h (duration adjusted according to molecular weight). PVDF membrane was blocked and then incubated with primary antibodies (Bax, Bcl-2, Collagen I, Collagen III, PI3K, p-PI3K, AKT and p-AKT, all 1:1,000 dilution), followed by incubation with secondary antibody (A0208, Beyotime, Shanghai, China, diluted 1:1,000). Finally, the membrane was developed using ECL substrate (P0018S, Beyotime, China) and visualized using a chemiluminescence imaging system (Tanon-4200, Tanon, Shanghai, China).

### 2.5 Statistical analysis

Image analysis was performed using ImageJ software (National Institutes of Health, Bethesda, MD, United States) to quantify specific features and staining intensities in the acquired histological and immunohistochemical images. GraphPad Prism 8.0 (GraphPad Software Inc., California, United States) was used for statistical analysis. For comparisons between two independent samples, data distribution normality was assessed using the D’Agostino–Pearson omnibus test, followed by evaluation of variance homogeneity using the Brown–Forsythe test. When both assumptions were met, an unpaired t-test was applied; otherwise, the Mann–Whitney U test was used. For comparisons among three or more groups, the same assumption tests were first conducted. If the data satisfied the assumptions of normality and homogeneity of variances, one-way ANOVA was performed, followed by Tukey’s multiple comparisons test as the *post hoc* analysis. If the assumptions were not met, the Kruskal–Wallis test was used, followed by Dunn’s *post hoc* test. All data are expressed as mean ± standard deviation ( $\bar{x}$ ± SD), from at least three independent biological replicates per experimental group. Statistical significance was defined as P < 0.05.

## 3 Results

### 3.1 PHSD metabolites characterization

A total of 133 metabolites were identified. The most abundant metabolites included glycyrrhizic acid, cornuside, liquiritin, isoliquiritigenin, quinic acid. Cardioactive metabolites such as aconitine, hypaconitine, and ginsenosides were also detected (Supplementary File 3, Table C1). The fingerprint spectrum of metabolite provided in Supplementary File 3, Table C2.

### 3.2 Network analysis of PHSD for HF

**FIGURE 2:**
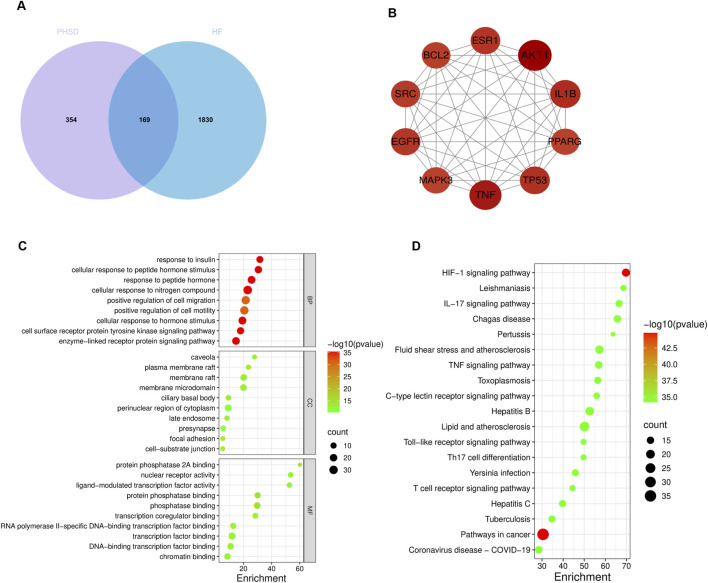
Network analysis of PHSD and heart failure. **(A)** Venn diagram illustrating overlapping targets between PHSD and heart failure. **(B)** PPI network of core targets. **(C)** Top 10 significantly enriched GO terms categorized by biological process (BP), cellular component (CC) and molecular functions (MF). **(D)** Top 20 significantly enriched KEGG pathways.

A total of 44 metabolites and 633 targets were selected for PHSD (Supplementary File 4, Tables D1, D2). Subsequent searches of GeneCards databases yielded and selected top 2,000 targets ranked by relevance score of HF-related targets (Supplementary File 4, Table D3). Analysis revealed 169 common targets shared between PHSD and HF (Figure 2A). Protein-protein interaction (PPI) network analysis identified AKT1, TNF, IL1B, TP53, ESR1, EGFR, PPARG, BCL2, SRC, MAPK3 as the top ten key targets (Figure 2B), suggesting their potential mediation of PHSD’s therapeutic effects in HF.

GO enrichment analysis identified 1,391 biological processes (BP), 83 cellular components (CC) and 114 molecular functions (MF) (Supplementary File 4, Tables D4-D5). BP terms were primarily associated with cellular response to nitrogen compound, response to peptide hormone and cellular response to hormone stimulus. CC terms mainly comprised membrane raft, membrane microdomain and plasma membrane raft. MF terms were predominantly related to phosphatase binding, protein phosphatase binding and protein phosphatase 2A binding. The top 10 enriched terms in each category are displayed in Figure 2C. KEGG analysis revealed 187 significant pathways (P < 0.05) (Supplementary File 4, Table D7), with the cancer, HIF-1 signaling, and lipid and atherosclerosis pathways being the most significantly associated with PHSD’s therapeutic effects against HF (Figure 2D).

### 3.3 Poge heart-saving decoction improves cardiac function in ISO-induced HF mice

**FIGURE 3:**
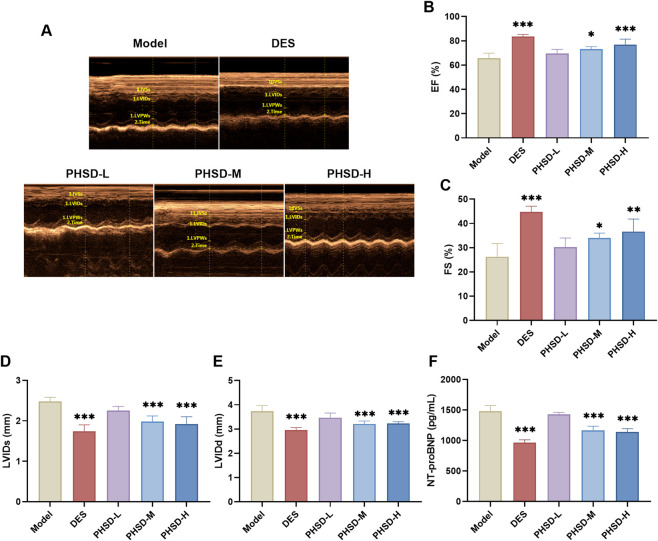
PHSD improved cardiac function. **(A)** Representative echocardiography images across groups (n = 5). **(B)** EF. **(C)** FS. **(D)** LVIDs. **(E)** LVIDd. **(F)** Serum NT-proBNP expression levels (n = 3). *P < 0.05, **P < 0.01, ***P < 0.001 versus model group.

In the model group, LVEF was significantly decreased, accompanied by elevated NT-proBNP levels, a well-established biomarker and prognostic indicator of HF (Cao et al., 2019), confirmed successful HF model establishment (Nanda et al., 2023). Compared with the model group, the DES, PHSD-M and PHSD-H groups showed significantly decreased LVIDs and LVIDd, accompanied by increased EF and FS (P < 0.05, Figures 3A–E). Furthermore, N-terminal pro b-type natriuretic peptide (NT-proBNP) expression was markedly reduced in the DES, PHSD-M and PHSD-H groups (P < 0.01), whereas the PHSD-L group showed no significant alteration (P > 0.05, Figure 3F). Notably, PHSD demonstrated efficacy comparable to deslanoside, indicating significant therapeutic potential. The medium dose (46.8 g/kg) exhibited the most pronounced effect. The clinical application of deslanoside is limited due to its adverse side effects (Fisch and Knoebel, 1985). Combined with previous reports (Zhan et al., 2023), these findings suggest that PHSD represents an effective and safe therapeutic alternative for HF.

### 3.4 Poge heart-saving decoction attenuates ISO-induced myocardial apoptosis in mice

**FIGURE 4:**
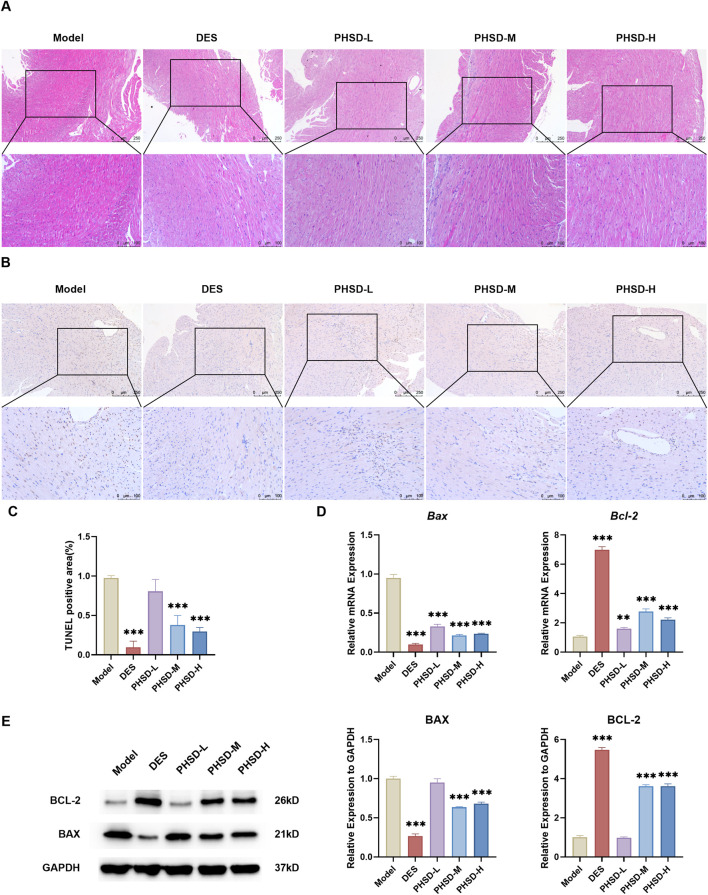
PHSD attenuates ISO-induced myocardial apoptosis in mice (n = 3). **(A)** Representative HE-stained images of myocardial tissues (magnification: ×200; scale bar = 100 μm). **(B)** Representative TUNEL-stained images of myocardial tissues (magnification: ×200; scale bar = 100 μm). Brown-stained nuclei represent apoptotic cardiomyocytes. **(C)** Quantification of TUNEL-positive nuclei across groups. **(D)** mRNA expression of *Bax* and *Bcl-2* in myocardial tissues. **(E)** Protein expression levels of Bax and Bcl-2 in myocardial tissues. *P < 0.05, **P < 0.01, ***P < 0.001 versus model group.

HE staining revealed disorganized myocardial cell arrangement in the model group, showing evident cytoplasmic dissolution and myofibrillar fragmentation. In contrast, both deslanoside and PHSD treatment significantly ameliorated myocardial tissue damage (Figure 4A). TUNEL staining revealed a significant increase in myocardial apoptosis in the model group. The DES, PHSD-M and PHSD-H groups exhibited markedly reduced ISO-induced myocardial apoptosis (P < 0.001), while the PHSD-L group showed no statistically significant improvement (P > 0.05, Figures 4B,C). The mRNA expression of Bax was significantly reduced in the DES and PHSD groups compared with the model group (P < 0.001), accompanied by upregulation of Bcl-2 (P < 0.01, Figure 4D). Similarly, Western blot analysis confirmed decreased Bax protein expression and increased Bcl-2 expression in the DES, PHSD-M and PHSD-H groups compared to the model group (P < 0.001, Figure 4E). These findings revealed that PHSD inhibits the myocardial apoptosis.

### 3.5 Poge heart-saving decoction ameliorates ISO-induced myocardial fibrosis in mice

**FIGURE 5:**
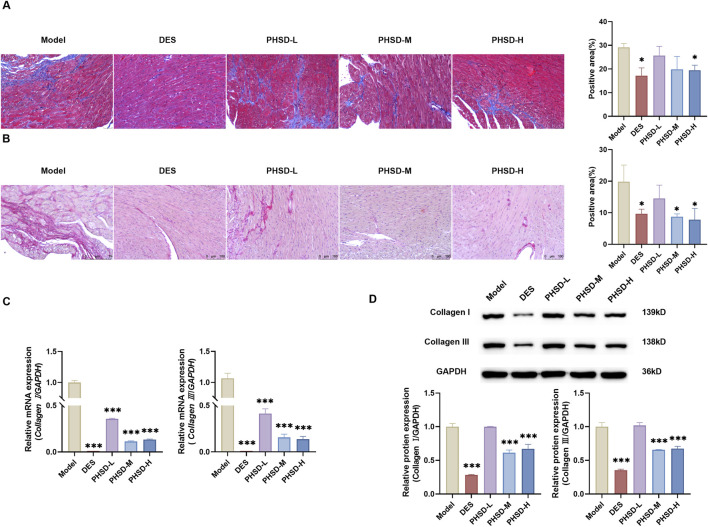
PHSD alleviates ISO-induced cardiac fibrosis (n = 3). **(A)** Representative images and quantitative analysis of Masson staining in myocardial tissue. Blue staining indicates collagen deposition (magnification: ×200, scale bar = 50 μm). **(B)** Representative images and quantitative analysis of Sirius Red staining in myocardial tissue. Pink staining indicates collagen deposition (magnification: ×200, scale bar = 100 μm). **(C)** *Collagen I* and *Collagen III* mRNA expression levels. **(D)** Protein expression levels of Collagen I and Collagen III in cardiac tissue. *P* < 0.05, *P < 0.01, **P < 0.001 versus model group.

Cardiac fibroblast proliferation and extracellular matrix (ECM) deposition constitute the pathological basis of myocardial fibrosis and significantly contribute to HF progression (Travers et al., 2016). Masson staining revealed extensive blue collagen deposition in myocardial tissues at the model group, indicating pronounced myocardial fibrosis. Compared with the model group, the DES, PHSD-M and PHSD-H groups demonstrated significantly reduced in myocardial collagen deposition. The PHSD-L group showed only marginal improvement (Figure 5A). Sirius Red staining revealed significantly increased collagen deposition and in the model group, which was reduced by PHSD treatment in a dose-dependent manner (Figure 5B). At the molecular level, the mRNA expression levels of Collagen I and Collagen III were significantly elevated in the model group, but markedly decreased in both the DES and PHSD groups (P < 0.001, Figure 5C). Western blot analysis further confirmed that PHSD attenuated ISO-induced upregulation of Collagen I and Collagen III in the medium- and high-dose groups (P < 0.001, Figure 5D). These results demonstrated that PHSD partially reversed ISO-induced collagen deposition.

### 3.6 Poge heart-saving decoction alleviates myocardial fibrosis through PI3K/AKT pathway inhibition

**FIGURE 6:**
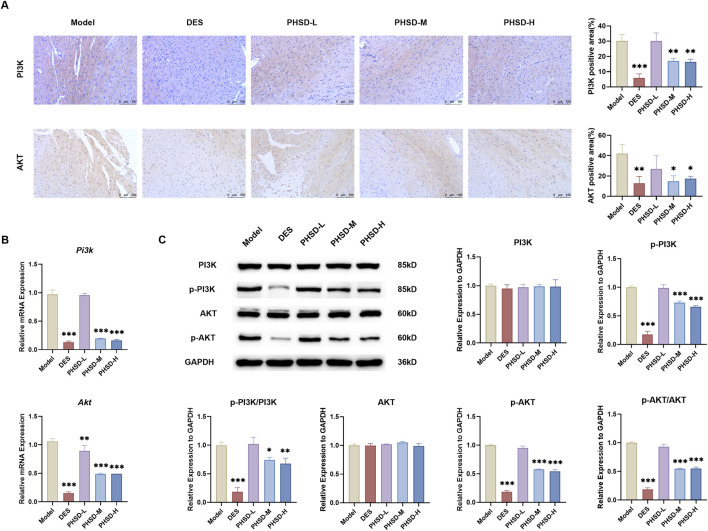
PHSD inhibits the PI3K/AKT pathway in ISO-induced heart failure model mice (n = 3). **(A)** Representative images and quantitative analysis of immunohistochemical staining for PI3K and AKT in myocardial tissue (magnification: ×200, scale bar = 100 μm). Brown-stained areas represent regions of positive expression. **(B)** Relative mRNA expression levels of *Pi3k* and Akt in myocardial tissue. **(C)** Representative protein expression levels of PI3K/AKT pathway in myocardial tissue. *P < 0.05, **P < 0.01, ***P < 0.001 versus model group.

According to the findings of the network analysis, most key targets are associated with the regulation of PI3K/AKT signaling. Furthermore, research indicates that the PI3K/AKT signal regulates upstream of HIF-1, and inhibiting the PI3K/AKT/HIF-1 pathway improves cardiac function and reduces myocardial fibrosis in HF mice (Li et al., 2025). So, the PI3K/AKT pathway was investigated. IHC staining revealed that the expressions of PI3K and AKT were increased in model group. PHSD-M, PHSD-H and deslanoside could downregulate PI3K and AKT expression (P < 0.05), while the PHSD-L showed no significant changes (P > 0.05, Figure 6A). RT-qPCR analysis showed that Pi3k and Akt mRNA expression levels were significantly reduced in the DES, PHSD-M, and PHSD-H groups, while no significant changes were observed in the PHSD-L group (P > 0.05, Figure 6B). WB analysis further validated these findings. Although total PI3K and AKT expression showed no significant differences among groups, the levels of p-PI3K, p-AKT, p-PI3K/PI3K and p-AKT/AKT were significantly decreased in the DES, PHSD-M, and PHSD-H groups, suggesting that PHSD could inhibit the phosphorylation of PI3K and AKT (P < 0.01). By contrast, the PHSD-L group exhibited no significant differences (P > 0.05, Figure 6C).

## 4 Discussion

This study provides the comprehensive investigation of the mechanisms underlying PHSD’s therapeutic effects on HF. While previous studies have attempted to explore cardioprotective mechanisms of PHSD, they were limited to clinical observations (Huang et al., 2021; Wu et al., 2022; Zhan et al., 2023; Zhou et al., 2024), preliminary experiments (Liang and Chen, 2006; Liu et al., 2020), or component-based research (Zhang et al., 2024). Our results indicate that PHSD alleviates ISO-induced cardiomyocyte apoptosis and myocardial fibrosis, with a mechanism involving the PI3K/AKT pathway. These findings establish a theoretical foundation for its clinical application.

Network analysis indicated that PHSD may mediate its therapeutic effects through targeting AKT1, TNF, and BCL2. Accumulating evidence suggests that these targets play critical roles in myocardial apoptosis and fibrosis progression (Korshunova et al., 2021; Qin W. et al., 2021; Al-Botaty et al., 2022). GO enrichment analysis indicated that the therapeutic mechanisms of PHSD in HF may involve the regulation of hormone responses, membrane-associated processes, and phosphatase binding. These functions strongly associated with apoptosis and protein phosphorylation. KEGG enrichment analysis suggested that therapeutic effects of PHSD on HF may be mediated through modulation of the pathways related to cell survival and apoptosis. ISO activates the β-AR signaling pathway excessively, thereby activating the PI3K/AKT1 pathway. The activated AKT1 promotes proliferation and activation of cardiac fibroblasts, leading to collagen deposition and myocardial fibrosis. AKT1 also participates in regulating inflammatory responses, including promoting the expression of pro-inflammatory cytokines such as TNF-α, thereby exacerbating myocardial inflammation (Jiao et al., 2025). These processes collectively drive cardiac structural remodeling and functional deterioration, ultimately leading to the onset and progression of heart failure. In injured cardiomyocytes, AKT (particularly p-AKT1) is abnormally activated and correlates with increased release of cardiac enzymes and elevated apoptosis rates. Intervention with bellidifolin inhibits cardiomyocyte apoptosis by downregulating the abnormally elevated p-AKT1/AKT1 ratio (Li et al., 2022). In this study, PHSD downregulated Pi3k and Akt mRNA expression as well as p-PI3K/PI3K and p-AKT/AKT in the HF mice, indicating that PHSD treatment inhibited the PI3K/AKT pathway.

PHSD was developed by a renowned TCM expert Li Ke, consisting of Fuzi (Aconite Tuber), Ganjiang (Ginger Rhizome), Hongshen (Red Ginseng), Shanzhuyu (Cornus), Gancao (Licorice), Longgu (Ossa Draconis), Muli (Oyster) and Cishi (Magnetitum). In TCM theory, PHSD demonstrates therapeutic properties of consolidating Yin and preserving Yang, stabilizing vital essence while preventing its dissipation, which is commonly used for the treatment of critically ill patients with life-threatening conditions (Xie et al., 2020). In this formulation, Fuzi and Ganjiang act as the principal herbs for restoring and enhancing cardiac function. Gancao counteracts and mitigates the potential toxicity of Fuzi. Hongshen, a specially processed form of ginseng, subsequently improves cardiac performance when combined with Fuzi. These four herbs constitute the renowned “Renshen Sini Decoction,” which is widely used in TCM for treating critical conditions (Zhang J. et al., 2025). Shanzhuyu is described as “the foremost herb for rescuing collapse” in YiXue ZhongZhong CanXi Lu (医学衷中参西录) (Zhang, 2016). This book maintains that substantial doses of Shanzhuyu function to protect Yuanqi (original Qi), thereby effectively counteracting collapse. In TCM philosophy, Yuanqi represents the body’s fundamental vitality, and its depletion constitutes a critical determinant of mortality in severe conditions. Longgu, Muli and Cishi exhibit potent sedative and anchoring properties, enabling them to suppress rebellious Qi and consolidate Yuanqi. When combined with Shanzhuyu, these herbs act synergistically to prevent Yuanqi dispersion, stabilizing vital energy and ensuring patient safety during critical illness (Li et al., 2013). Consequently, PHSD not only ameliorates HF but also demonstrates therapeutic efficacy in various critical conditions including severe pneumonia (Xie et al., 2020), pulmonary heart disease (Wu et al., 2022), sepsis (Wang et al., 2025) and septic shock (Huang et al., 2021), which embodies the TCM principle of “treating different diseases with the same method” (yi bing tong zhi) (Zheng et al., 2015).

The high-dose application of Fuzi (Aconite Tuber) constitutes a defining characteristic of PHSD. Modern pharmacological studies have confirmed that Fuzi exhibits significant cardiotonic effects, corresponding to the TCM concept of “restoring yang to rescue from collapse.” Research has demonstrated that Aconite Tuber enhances cardiac function through multiple mechanisms including regulation of myocardial energy metabolism, suppression of inflammatory responses, inhibition of myocardial apoptosis and reduction of water-sodium retention (Zhang S. et al., 2025). The high-dose inclusion of Cornus represents another distinctive feature of this formulation. Jiang et al. confirmed that Cornus exerts cardioprotective effects and ameliorates cardiac ischemia-reperfusion injury (Jiang et al., 2011), potentially through reducing myocardial polymorphonuclear leukocyte infiltration and inhibiting myeloperoxidase activation. Cornus is primarily recognized for its antidiabetic properties (Dzydzan et al., 2019). Notably, current HF treatment guidelines (Heidenreich et al., 2022) recommend sodium-glucose cotransporter-2 (SGLT2) inhibitors (SGLT2i) as first-line therapy, demonstrating convergence between TCM and Western medicine (Packer, 2023). UHPLC-MS/MS was used to identify the components of PHSD, and glycyrrhizic acid, cornuside, liquiritin, isoliquiritigenin and quinic acid were found to show relatively high abundances. Previous studies have demonstrated that these ingredients may exert cardioprotective effects through anti-inflammatory and antioxidant mechanisms (Haleagrahara et al., 2011; Jiang et al., 2011; Tang et al., 2022; Liang et al., 2023), as well as by enhancing myocardial energy metabolism (Dong et al., 2022). Additionally, aconitine, hypaconitine and ginsenoside, known for their positive inotropic effects and ability to improve cardiac function, were detected in PHSD. These components may represent potential active substances in PHSD for alleviating HF, requiring further experimental investigation.

While this study explored certain mechanisms underlying PHSD’s therapeutic effects on HF and yielded promising results, several limitations should be acknowledged. First, this experiment did not employ inhibitors to verify whether the anti-HF effect of PHSD is mediated through inhibition of the PI3K/AKT pathway. Second, the investigation focused primarily on local tissue structure and molecular expression, without exploration of downstream PI3K/AKT signaling, leaving the underlying mechanisms incompletely elucidated. Considering the multi-component and multifaceted mechanisms of TCM in disease regulation, further experimental studies are required to elucidate the potential targets and key components of PHSD treating HF. This will be the focus of our future research.

## 5 Conclusion

In this study, PHSD exhibits therapeutic potential for HF. It is confirmed to inhibit cardiomyocyte apoptosis and attenuation of myocardial fibrosis. These anti-apoptotic and anti-fibrotic effects are mainly mediated via suppression of the PI3K/AKT signaling pathway. These findings reveal part of the mechanism by which PHSD treats heart failure, providing laboratory evidence for its clinical application.

## Data Availability

The original contributions presented in the study are publicly available. This data can be found here: https://ngdc.cncb.ac.cn/omix/release/OMIX015605.
