# Supplementary material for: Poge heart-saving decoction meliorates heart failure by suppressing apoptosis and fibrosis via regulation of the PI3K/AKT pathway
Source: Front Pharmacol. 2026 Mar 25;17:1748420. doi: 10.3389/fphar.2026.1748420 (PMC13058608; doi:10.3389/fphar.2026.1748420)
Supplement: Supplementary file 1 [file Supplementaryfile5.pdf]

重庆医科大学动物实验伦理审查同意书

批准编号:IACUC-CQMU-2024-0330

项目名称:破格救心汤治疗射血分数保留型心衰的研究

项目来源：重庆市科卫联合项目（2020ZY023975）

项目负责人:杜磊

负责人单位:重庆市中医院

申请日期:2024年03月19日

批准日期:2024年04月07日

本项目所涉及的动物实验方案经过重庆医科大学实验动物管理和使用委员会（IACUC-CQMU）审查，符合动物保护、动物福利和伦理原则，符合国家实验动物福利伦理的相关规定，同意按此方案进行实验。

重庆医科大学实验动物管理和使用委员会

2024年04月07日

IACUC Approval Certification

Approval number:IACUC-CQMU-2024-0330

Project title:Study on the Treatment of Heart Failure with Preserved Ejection Fraction by Poge Jiuxin Recipe

Funding source:Chongqing Science and Health Joint Program (2020ZY023975)

Principal investigator:Lei Du

Department:Chongqing Hospital of Traditional Chinese Medicine

Application date:2024-03-19

Approval date:2024-04-07

All animal procedures to be employed in the project was approved by Institutional Animal Care and Use of Chongqing Medical University (IACUC-CQMU).

Jun Yuan

Jun Yuan， MD
Chair, IACUC
Chongqing Medical University
