# Supplementary material for: Poge heart-saving decoction meliorates heart failure by suppressing apoptosis and fibrosis via regulation of the PI3K/AKT pathway
Source: Front Pharmacol. 2026 Mar 25;17:1748420. doi: 10.3389/fphar.2026.1748420 (PMC13058608; doi:10.3389/fphar.2026.1748420)
Supplement: Supplementary file 3 [file Supplementaryfile1.docx]

Technical Requirements for Quality Control and Standard-Setting of Traditional Chinese Medicine (TCM) Formula Granules

1. Prepared Aconite Root (Fuzi) Formula Granules (XLS-09-TP-3629/R05)

**1. Purpose**

To establish the quality standard for Prepared Aconite Root (Fuzi) formula granules to ensure product quality.

**2. Applicable Scope**

This standard applies to the quality inspection of Prepared Aconite Root (Fuzi) formula granules.

**3. Responsibility**

The Quality Management Department is responsible for drafting, reviewing, and approving this standard. Quality inspection personnel shall strictly implement this standard.

**4. Content**

**4.1 Basic Information**

Product Code (Specification 1): 0900110106

Product Code (Specification 2): 10001101100

**4.2 Sampling and Testing Methods**

Sampling Method: Sampling Procedure XLS-09-OP-2910

Testing Method: Inspection Procedure for Baifu Pian Formula Granules XLS-09-OP-3745

**4.3 Standard Specifications**

**Description:**

This product is a formula granule prepared from *Baifu Pian*, a processed variety of the daughter root (*Fuzi*) of *Aconitum carmichaeli* Debx. (Family: Ranunculaceae).

**Processing:**

Prepared in accordance with the *Processing* section of *Baifu Pian* in *Chinese Pharmacopoeia* (2020 Edition, Vol. I).

**Manufacturing Process:**

Refer to *Baifu Pian Formula Granule Manufacturing Procedure* (XLS-08-TP-0007).

**Characteristics:**

Grayish-white to yellowish-white granules; slight odor; mild taste.

**Identification**

Weigh 2 g of *Baifu Pian* formula granules, add 30 ml of ether and 5 ml of ammonia solution, shake for 20 minutes, and filter. Transfer the filtrate to a separatory funnel, add 20 ml of 0.25 mol/L sulfuric acid, and extract. Separate the acidic layer and test by UV spectrophotometry (Procedure XLS-09-OP-2894) using water as blank. The spectrum should show maximum absorption at 231 ± 2 nm and 274 ± 2 nm.

**Tests**

Particle Size: Determined according to Particle Size Determination Procedure (XLS-09-OP-2874).

Particle size shall not exceed 15%.

Moisture: Determined by Moisture Determination Procedure (XLS-09-OP-2873), Method II.

Moisture shall not exceed 8.0%.

Solubility: Dissolve one sachet (10 g for multi-dose packaging) in 200 ml of hot water, stir for 5 minutes, and observe immediately. The solution shall dissolve completely or be slightly turbid, with no scorched particles or foreign matter.

**Content Uniformity / Fill Weight Variation:**

Tested according to Fill Weight Variation Determination Procedure (XLS-09-OP-2900).

For single-dose packaging: outliers shall not exceed 2 samples, and no single sample shall exceed twice the allowable limit.

For multi-dose packaging: the average fill weight shall not be lower than the labeled amount, and no single unit shall be less than 97% of the labeled amount. If one fails, retest three additional units—all must comply.

**Assay**

**Method:**

High-Performance Liquid Chromatography (HPLC) according to *HPLC Operating Procedure* (XLS-09-OP-2895).

Determination of Diester-type Alkaloids

Chromatographic Conditions:

Stationary phase: Octadecylsilyl-bonded silica gel (C18).

Mobile phase:

A: Acetonitrile–tetrahydrofuran (25:15)

B: 0.1 mol/L ammonium acetate (add 0.5 ml glacial acetic acid per 1000 ml)

Gradient elution:

| Time (min) | Mobile Phase A (%) | Mobile Phase B (%) |
| --- | --- | --- |
| 0–48 | 15 → 26 | 85 → 74 |
| 48–49 | 26 → 35 | 74 → 65 |
| 49–58 | 35 | 65 |
| 58–65 | 35 → 15 | 65 → 85 |

Detection wavelength: 235 nm.

**Preparation of Reference Solution:**

Weigh about 5.5 mg of *Aconitum* diester-type alkaloid reference extract (containing approximately 31.7% neoline, 30.0% mesaconitine, and 31.8% aconitine).

Dissolve in isopropanol–dichloromethane (1:1) and make up to 10 ml. Shake well.

Pipette 3 ml of this stock solution into a 100 ml volumetric flask, dilute to volume with isopropanol–dichloromethane (1:1), and shake well to obtain a working reference solution containing approximately 5 μg/ml of each alkaloid.

**Preparation of Test Solution:**

Grind sample to fine powder, weigh 2 g, add 3 ml ammonia solution, and 50 ml isopropanol–ethyl acetate (1:1).

Ultrasonicate (300 W, 40 kHz, below 25 °C) for 30 min, cool, restore lost weight, shake, and filter.

Evaporate 25 ml of filtrate under reduced pressure below 40 °C to dryness, dissolve the residue in 3 ml isopropanol–chloroform (1:1), filter, and use the filtrate as the test solution.

**Determination:**

Inject 10 μl each of reference and test solutions into the HPLC system and record chromatograms.

**Content Requirement:**

The total content of diester-type alkaloids—calculated as aconitine (C₃₄H₄₇NO₁₁), mesaconitine (C₃₃H₄₅NO₁₀), and neoline (C₃₃H₄₅NO₁₁)—shall not exceed 0.010%.

**Extractives**

Determined by Extractives Determination Procedure (XLS-09-OP-2877) using the hot extraction method.

The ethanol-soluble extractives shall not be less than 8.4%.

**Microbial Limits**

Determined according to *Microbial Limit Determination Procedure* (XLS-09-OP-2893).

| Microorganism | Limit |
| --- | --- |
| Total aerobic count | 10³ CFU/g (max 2000 CFU/g) |
| Total molds and yeasts | 10² CFU/g (max 200 CFU/g) |
| *Escherichia coli* | Absent |

**Functions and Indications**

Restores yang and rescues from collapse, reinforces fire and assists yang, dispels cold and alleviates pain.

Indicated for yang exhaustion with cold limbs and faint pulse, deficiency of heart yang with chest pain, cold vomiting and diarrhea, abdominal cold pain, kidney yang deficiency, impotence, cold uterus, cold edema, cold arthralgia, and cold-induced disorders.

**Dosage and Administration**

Used for prescription compounding; administer as directed by physician.

**Precautions**

Use with caution in pregnancy.

Contraindicated for concurrent use with *Pinellia ternata* (Banxia), *Trichosanthes kirilowii* (Gualou), *Fritillaria* spp. (Beimu), *Bletilla striata* (Baiji), and *Ampelopsis japonica* (Bailian).

**Specifications**

Specification 1: 0.5 g per sachet (equivalent to 6 g crude drug)

Specification 2: 100 g per sachet (equivalent to 1100 g crude drug)

**Packaging**

Aluminum-plastic composite film.

**Storage**

Sealed and stored in a cool, dry place.

**Shelf Life**

Three years.

1. Dried Ginger Formula Granules (YBZ-PFKL-2021050)

**[Source]** This product is a formula granule prepared from the processed and dried rhizome of *Zingiber officinale* Rosc. (family Zingiberaceae), in accordance with the main quality standards of the standard decoction.

**[Preparation Method]** Take 6000 g of *Zingiber officinale* Rosc. decoction pieces, add water and decoct. Filter, and concentrate the filtrate to obtain a clear extract (with a dry extract yield of 8.5%–16.5%). Dry (or dry and pulverize), add an appropriate amount of excipients, mix evenly, and granulate to obtain 1000 g of the finished product.

**[Characteristics]** The product appears as yellowish-white to brownish-yellow granules, possessing a slight odor and a pungent taste.

**[Identification]** Take 1 g of the sample, grind it finely, and dissolve it in 10 mL of water. Extract twice with 20 mL of ethyl acetate each time by shaking. Combine the ethyl acetate extracts, evaporate to dryness, and dissolve the residue in 1 mL of methanol as the test solution. Separately, take 1 g of the reference crude drug of *Zingiber officinale* and add 20 mL of ethyl acetate. Sonicate for 10 minutes and filter. Use the filtrate as the reference crude drug solution. Prepare a reference standard solution of 6-gingerol in methanol to contain 0.5 mg per mL. According to the method described under Thin-layer Chromatography (General Rule 0502, Chinese Pharmacopoeia 2020 edition), apply 5 μL of the test solution, and 6 μL each of the reference crude drug solution and the reference standard solution on the same silica gel G plate. Develop with a mobile phase consisting of petroleum ether (60–90 °C)–chloroform–ethyl acetate (2 : 1 : 1). Remove the plate, dry it in air, spray with vanillin–sulfuric acid reagent, and heat at 105 °C until spots are clearly visible. In the chromatogram of the test solution, a spot appears at the position corresponding to that in the chromatograms of both the reference crude drug and the reference standard solutions, showing the same color.

**[Characteristic Chromatogram]** Determine by High Performance Liquid Chromatography (HPLC) according to the method described under General Rule 0512 of the Chinese Pharmacopoeia (2020 edition).

**Chromatographic Conditions and System Suitability Test**

Use octadecylsilyl-bonded silica gel as the stationary phase. Use acetonitrile as mobile phase A and water as mobile phase B, and perform gradient elution according to the table below. The flow rate is 1.0 mL per minute, the column temperature is maintained at 40 °C, and the detection wavelength is set at 215 nm. The number of theoretical plates calculated based on the peak of 6-gingerol should not be less than 5000.

| Time (min) | Mobile phase A (%) | Mobile phase B (%) |
| --- | --- | --- |
| 0～5 | 15→35 | 85→65 |
| 5～25 | 35→70 | 65→30 |
| 25～40 | 70→90 | 30→10 |
| 40～50 | 90 | 10 |

**Preparation of Reference Solutions**

Weigh 0.5 g of the reference crude drug of *Zingiber officinale* (dried ginger), add 25 mL of methanol, and ultrasonicate (power 250 W, frequency 40 kHz) for 30 minutes. Shake well and filter. Use the successive filtrate as the reference crude drug solution.

Separately, accurately weigh appropriate amounts of 6-gingerol reference standard and hexahydrocurcumin reference standard. Dissolve each in methanol to prepare solutions containing 0.1 mg of each per mL, respectively. These are used as the reference standard solutions.

**Preparation of Test Solution**

An appropriate amount of the test sample was finely ground, and approximately 0.4 g was taken. The test solution was prepared following the same procedure as the “reference herbal solution.”

**Assay Method**

Precisely 10 µL of the reference solution and 10 µL of the test solution were injected into the liquid chromatograph for measurement. The chromatogram of the test solution should display five characteristic peaks, corresponding in retention time to the five characteristic peaks of the reference herbal solution. Peaks 3 and 4 should correspond to the retention times of the reference standards of tetrahydrocurcumin and 6-gingerol, respectively. The peak corresponding to 6-gingerol in the reference solution is designated as the S peak. The relative retention times of peaks 1, 2, and 5 to the S peak should fall within ±10% of the specified values. The specified relative retention times are: 0.18 (peak 1), 0.21 (peak 2), and 1.39 (peak 5).


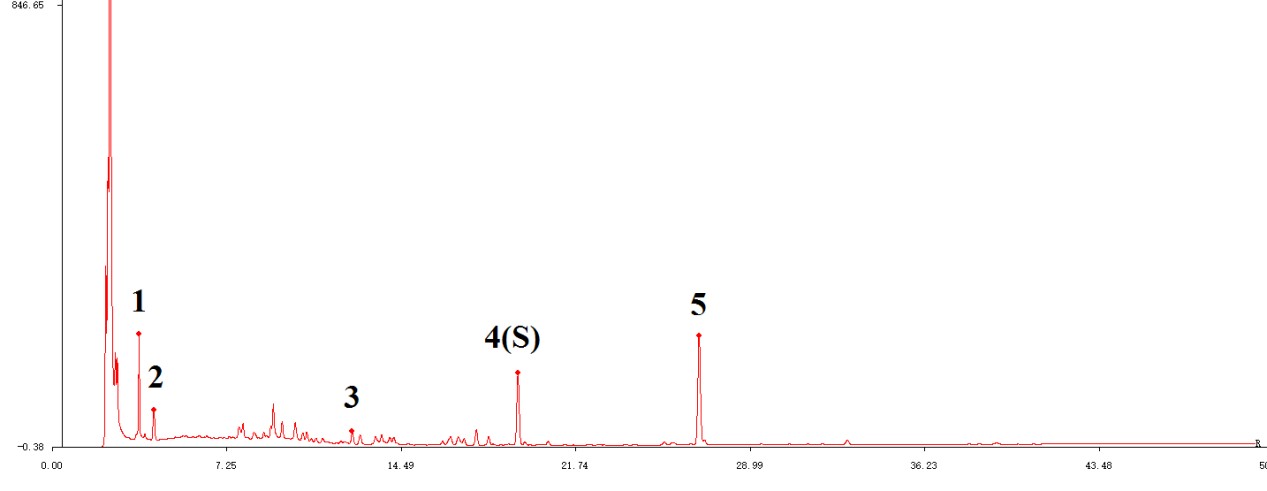


**Characteristic chromatogram peaks**

Peak 3: Hexahydrocurcumin; Peak 4 (S): 6-Gingerol; Peak 5: 6-Shogaol.

Column: Platisil ODS C18 (4.6 mm × 250 mm, 5 μm).

**[Inspection]**

Should comply with the general requirements for granules (Chinese Pharmacopoeia, 2020 edition, General Rule 0104).

**[Extractives]**

Determined according to the method for ethanol-soluble extractives (Chinese Pharmacopoeia, 2020 edition, General Rule 2201) using the hot extraction method with ethanol as the solvent. The content shall not be less than 14.0%.

**[Assay]**

Determined by high-performance liquid chromatography (Chinese Pharmacopoeia, 2020 edition, General Rule 0512).

**Chromatographic Conditions and System Suitability Test**

Use octadecylsilane-bonded silica gel as the stationary phase (column length 100 mm, inner diameter 2.1 mm, particle size 1.7 μm);

mobile phase: methanol–water–acetonitrile (5:55:40);

flow rate: 0.4 mL/min;

column temperature: 30 °C;

detection wavelength: 280 nm.

Theoretical plate number calculated based on the 6-gingerol peak should not be less than 5000.

**Preparation of Reference Solution**

Accurately weigh an appropriate amount of 6-gingerol reference standard, dissolve in methanol to make a solution containing 70 μg per mL.

**Preparation of Test Solution**

Grind an appropriate amount of the sample into powder. Accurately weigh about 0.25 g, place it in a stoppered conical flask, add precisely 20 mL of 75% methanol, stopper tightly, weigh, and ultrasonicate (power 250 W, frequency 40 kHz) for 30 minutes.

Cool, weigh again, and replenish any weight loss with 75% methanol. Shake well and filter. Use the subsequent filtrate as the test solution.

**Assay Procedure**

Accurately inject 1 μL of the reference solution and 0.5–1 μL of the test solution into the liquid chromatograph and determine.

Each 1 g of the granules contains 5.0 mg–13.0 mg of 6-gingerol (C₁₇H₂₆O₄).

**[Specification]**

Each 1 g of the formula granules is equivalent to 6 g of crude herbal material.

**[Storage]**

Sealed.

1. Red Ginseng (Hongshen) Formula Granule (XLS-09-TP-15342/CA00)

**1. Purpose**

To establish the quality standard for Red Ginseng Formula Granule.

**2. Scope of Application**

This standard applies to the quality inspection of Red Ginseng Formula Granule.

**3. Responsibility**

The Quality Management Department is responsible for the drafting, review, and approval of this standard. Quality control personnel shall strictly implement the requirements of this document.

**4. Content**

**4.1 Basic Information**

4.1.1 Material Codes

Extract code: H25019901

Granule code: H35019901

4.1.2 Reference Standards

Based on Chinese Pharmacopoeia (2020 Edition) and Red Ginseng Formula Granule Public Draft.

4.2 Sampling Method

According to the Sampling Procedure (Document No. XLS-QA-OP-0003).

4.3 Standard Specifications

4.3.1 Extract

**[Properties]**

This product is a light yellow to brownish-yellow powder; it has a slight odor, and tastes sweet and slightly bitter.

**[Identification]**

Weigh 0.5 g of the sample, grind finely, moisten with 0.5 ml of water, add 10 ml of water-saturated n-butanol, ultrasonicate for 30 minutes, and use the supernatant for testing. Separately, prepare a reference drug solution and reference substance solution as follows:

Reference drug solution: Take 0.5 g of Red Ginseng reference material, add 40 ml chloroform, reflux for 1 hour, discard the chloroform layer, dry the residue, add 25 ml water, reflux for 30 minutes, filter, concentrate the filtrate to near dryness, add 10 ml water-saturated n-butanol, and treat as above.

Reference substance solution: Prepare a mixed methanol solution containing 1 mg/ml each of Ginsenoside Rb1, Re, Rf, and Rg1.

Perform thin-layer chromatography (TLC) according to the TLC Operating Procedure (Document No. XLS-09-OP-2897). Spot 2–3 μl of the test solution, 2 μl of the reference drug solution, and 1 μl of the reference substance solution on the same silica gel G plate. Develop with a mobile phase consisting of chloroform–ethyl acetate–methanol–water (15:40:22:10, lower layer) below 10°C. After development, dry the plate, spray with 10% sulfuric acid–ethanol solution, heat at 105°C until spots appear clearly, and observe under daylight and UV light (365 nm). The chromatogram of the test solution should show spots corresponding in color and position to those of the reference drug and reference substances.

**[Characteristic Chromatogram]**

Determine by High-Performance Liquid Chromatography (HPLC) according to the HPLC Operating Procedure (Document No. XLS-09-OP-2895).

Chromatographic Conditions and System Suitability

Column: Octadecylsilane-bonded silica gel (150 mm × 2.1 mm, 1.6 µm)

Mobile phase: A: Acetonitrile; B: 0.01% phosphoric acid solution

Gradient program:

| Time (min) | A (%) | B (%) |
| --- | --- | --- |
| 0–9 | 21 | 79 |
| 9–12 | 21→28 | 79→72 |
| 12–32 | 28→33 | 72→67 |
| 32–38 | 33→40 | 67→60 |
| 38–47 | 40→59 | 60→41 |

Flow rate: 0.35 ml/min; Column temperature: 30°C; Detection wavelength: 203 nm

Theoretical plate number based on Ginsenoside Rg1 peak should not be less than 6000.

**Preparation of Reference Solution**

Take 1 g of Red Ginseng reference material, add 25 ml water, reflux for 30 minutes, cool, filter, concentrate to near dryness, add 15 ml 80% methanol, ultrasonicate (600 W, 40 kHz) for 30 minutes, cool, filter, and collect the filtrate as the reference drug solution. Prepare the reference substance solution according to the assay section.

**Preparation of Test Solution**

Same as described under Assay.

**Determination**

Inject 1 μl each of the reference and test solutions into the HPLC system and record chromatograms. The chromatogram of the test solution should exhibit 12 characteristic peaks corresponding to those of the reference drug chromatogram. Peaks 1–2 and 5 should correspond to the same retention times as their reference substances. Peak 5 (S) corresponds to Ginsenoside Rb1. Relative retention times of other peaks should be within ±10% of the specified values: 0.68 (Peak 3), 0.75 (Peak 4), 1.04 (Peak 6), 1.09 (Peak 7), 1.18 (Peak 8), 1.36 (Peak 9), 1.59 (Peak 10), 1.70 (Peak 11), 1.71 (Peak 12). The relative peak area of Peak 10 to Peak 1 shall not be less than 0.08.


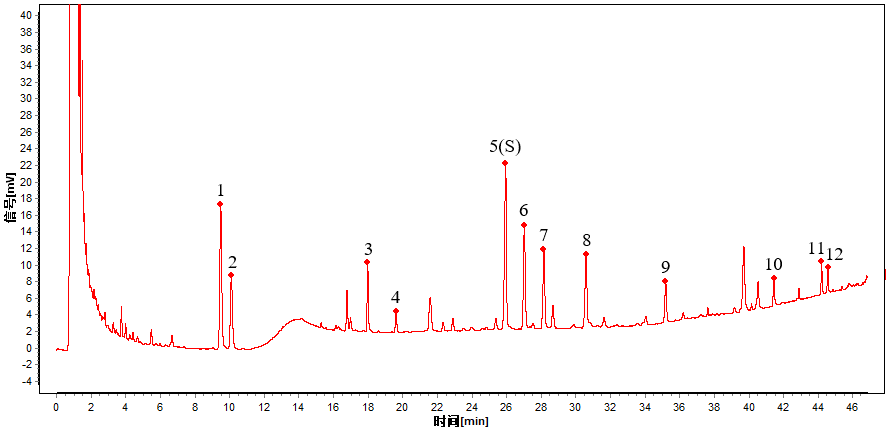


**Characteristic chromatogram peaks**

Peak 1: Ginsenoside Rg1; Peak 2: Ginsenoside Re; Peak 3: Ginsenoside Rf; Peak 5 (S): Ginsenoside Rb1; Peak 6: Ginsenoside Ro; Peak 10: Ginsenoside Rh4; Peak 11: Ginsenoside Rg3; Peak 12: 20(R)-Ginsenoside Rg3. Column: CORTECS T3 (2.1 mm × 150 mm, 1.6 µm).

**[Tests]**

**(1) Pesticide Residues (Organochlorine)**

Determine by Gas Chromatography (GC). Column: capillary column coated with 14% cyanopropylphenyl dimethylpolysiloxane (30 m × 0.32 mm × 0.25 µm). Detector: ECD (Ni⁶³). Programmed temperature: 60°C (0.5 min) → 170°C at 60°C/min → 220°C at 15°C/min (hold 5 min) → 240°C at 1°C/min → 280°C at 15°C/min (hold 5 min). Resolution between adjacent peaks ≥ 1.5. Limits: Pentachloronitrobenzene ≤ 0.1 mg/kg; Hexachlorobenzene ≤ 0.1 mg/kg; Heptachlor and Heptachlor Epoxide (sum) ≤ 0.05 mg/kg; Chlordane (cis-, trans-, and oxychlordane, sum) ≤ 0.1 mg/kg

**(2) Moisture**

Determined according to Moisture Determination Procedure (Document No. XLS-09-OP-2873, Method II). Limit: ≤ 6.5%.

**(3) Solubility**

Dissolve 10 g of sample in 200 ml of hot water, stir for 5 minutes, and observe. The sample should dissolve completely or appear slightly turbid; no carbonized or foreign matter shall be visible.

**(4) Extractives**

Determine by Hot Extraction Method (Document No. XLS-09-OP-2877) using ethanol as solvent. Not less than 24.2%.

**[Assay]**

Determine by HPLC according to HPLC Operating Procedure (Document No. XLS-09-OP-2895).

Chromatographic Conditions: Column: C18 (100 mm × 2.1 mm, 1.8 µm); Mobile phase: A: Acetonitrile, B: Water; Gradient program:

| Time (min) | A (%) | B (%) |
| --- | --- | --- |
| 0–10 | 19 | 81 |
| 10–14 | 19→29 | 81→71 |
| 14–17 | 29 | 71 |
| 17–23 | 29→40 | 71→60 |
| 23–24 | 40 | 60 |

Detection wavelength: 203 nm; Theoretical plate number ≥ 6000

Reference solution: Mixed methanol solution containing Ginsenoside Rg1 150 μg/ml, Re 60 μg/ml, Rb1 170 μg/ml.

Test solution: 0.5 g sample, 50 ml 80% methanol, ultrasonicate 30 min, cool, filter, make up to 5 ml. Inject 1 μl for analysis.

Content per gram: Total Rg1 + Re: 1.8–8.3 mg; Rb1: 1.5–8.0 mg

**[Microbial Limit Test]**

Total aerobic bacteria ≤ 10³ CFU/g (Max 2000 CFU/g); Total molds and yeasts ≤ 10² CFU/g (Max 200 CFU/g); Escherichia coli: Not detected

**4.3.2 Granule**

**[Properties]**

Light yellow to brownish-yellow granules; slight odor; sweet and slightly bitter taste.

**[Tests]**

Particle Size: ≤ 6.0% (XLS-09-OP-2874)

Moisture: ≤ 6.5% (XLS-09-OP-2873, Method II)

Solubility: Dissolve 10 g sample in 200 ml hot water, stir for 5 minutes, should be fully dissolved or slightly turbid; no carbonized or foreign matter.

1. Cornus Officinalis Formula Granules (YBZ-PFKL-2021110)

**[Source]**

This product is prepared from the dried ripe pulp of *Cornus officinalis* Sieb. et Zucc. (family *Cornaceae*), which is processed and manufactured into formula granules according to the main quality indicators of the corresponding decoction.

**[Preparation]**

Take 1200 g of *Cornus officinalis* decoction pieces, add water for decoction, filter, and concentrate the filtrate to a clear extract (dry extract yield: 42%–60%). Add an appropriate amount of excipients, dry (or dry and pulverize), then mix with additional excipients, and granulate to obtain 1000 g of product.

**[Properties]**

The product appears as light brown to reddish-brown granules; it has a slight odor and a sour, astringent, slightly bitter taste.

**[Identification]**

Weigh 1 g of the product, grind finely, add 10 ml of anhydrous ethanol, and ultrasonicate for 15 minutes. Filter, evaporate the filtrate to dryness, dissolve the residue in 1 ml of anhydrous ethanol—this is the test solution.

Take 1.5 g of *Cornus officinalis* reference herbal material, add 50 ml of water, boil for 30 minutes, filter, evaporate the filtrate to dryness, and dissolve the residue in 10 ml of anhydrous ethanol—this is the reference herbal solution. Prepare a reference substance solution by dissolving morroniside reference standard in anhydrous ethanol to make a solution containing 1 mg/ml. Perform the test according to the TLC method (Chinese Pharmacopoeia 2020, General Rule 0502). Spot 5 µl each of the three solutions on the same silica gel G TLC plate. Use ethyl acetate–ethanol–glacial acetic acid (50:10:1) as the developing solvent. After development, dry, spray with 5% vanillin–sulfuric acid reagent, and heat at 105 °C until spots appear clearly. In the chromatogram of the test solution, a purplish-red spot should appear at the same position as that in the reference substance chromatogram; other spots should correspond in color and position to those in the reference herbal chromatogram.

**[Characteristic Chromatogram]**

Determine according to the HPLC method (Chinese Pharmacopoeia 2020, General Rule 0512).

**Chromatographic conditions and system suitability test**

Stationary phase: Octadecylsilyl-bonded silica gel (column 150 mm × 2.1 mm, 1.7 µm) Mobile phase: A: Acetonitrile, B: 0.2% phosphoric acid solution.

Gradient elution:

| Time (min) | Mobile phase A (%) | Mobile phase B (%) |
| --- | --- | --- |
| 0~3 | 2→8 | 98→92 |
| 3~9 | 8→15 | 92→85 |
| 9~13 | 15→23 | 85→77 |
| 13~19 | 23→100 | 77→0 |
| 19.1~24 | 2 | 98 |

Flow rate: 0.2 ml/min; column temperature: 25 °C; detection wavelength: 260 nm.

Theoretical plate number calculated based on the morroniside peak should not be less than 10,000.

**Preparation of reference material solutions**

Take 0.2 g of *Cornus officinalis* reference herbal material, place in a conical flask with a stopper, add 10 ml of water, reflux for 60 minutes, cool, centrifuge, and collect the supernatant. Dilute to 20 ml with methanol, mix, and filter. Use the filtrate as the reference herbal solution. Separately, prepare a mixed reference substance solution by dissolving gallic acid, 5-hydroxymethylfurfural, loganin, and morroniside in 80% methanol to make a solution containing 20 µg/ml gallic acid, 20 µg/ml 5-hydroxymethylfurfural, 80 µg/ml loganin, and 100 µg/ml morroniside.

**Preparation of test solution**

Weigh approximately 0.1 g of the product, place in a conical flask with a stopper, add 20 ml of 80% methanol, ultrasonicate (500 W, 40 kHz) for 30 minutes, cool, mix well, and filter. Use the filtrate for testing.

**Determination method**

Inject 1 µl each of the reference and test solutions into the HPLC system and record the chromatograms. The test chromatogram should display 5 characteristic peaks corresponding to those in the reference herbal chromatogram, 4 of which should match the retention times of the corresponding reference substances. The peak corresponding to morroniside is designated as the S peak. Calculate the relative retention time of peak 5 to the S peak (should be within ±10% of 1.37), and the relative peak area of peak 1 to the S peak (should be within 0.35–3.00).


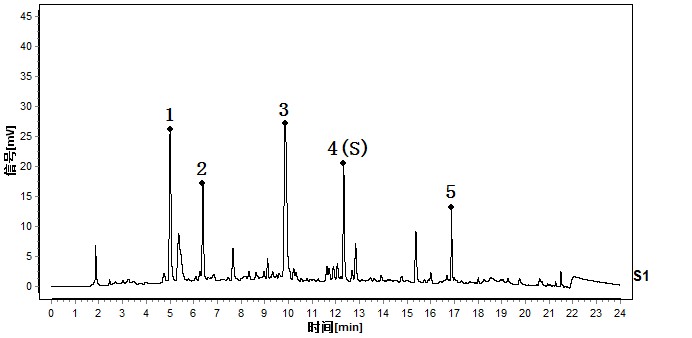


**Characteristic chromatogram peaks**

Peak 1: Gallic acid, peak 2: 5-Hydroxymethylfurfural, Peak 3: Loganin, Peak 4 (S): Morroniside, Column: BEH C18 (2.1 mm × 150 mm, 1.7 µm)

**[Tests]**

**Heavy metals and harmful elements**

Determine according to Chinese Pharmacopoeia 2020, General Rule 2321.

Limits: Pb ≤ 5 mg/kg; Cd ≤ 1 mg/kg; As ≤ 2 mg/kg; Hg ≤ 0.2 mg/kg; Cu ≤ 20 mg/kg.

Other requirements shall conform to the general provisions for granules (Chinese Pharmacopoeia 2020, 0104).

**Extractives**

Weigh about 2 g of finely powdered sample, add 100 ml ethanol, and determine by the hot ethanol-soluble extractives method (Chinese Pharmacopoeia 2020, 2201). The extractives should not be less than 30.0%.

**[Assay]**

Determine according to the HPLC method (Chinese Pharmacopoeia 2020, 0512).

**Chromatographic conditions**

Stationary phase: Octadecylsilyl-bonded silica gel.

Mobile phase:

A: Acetonitrile

B: 0.3% phosphoric acid solution

Flow rate: 0.3 ml/min; column temperature: 35 °C; detection wavelength: 240 nm.

Theoretical plate number based on morroniside should not be less than 10,000.

| Time (min) | Mobile phase A (%) | Mobile phase B (%) |
| --- | --- | --- |
| 0~4 | 7 | 93 |
| 4~13.5 | 7→20 | 93→80 |
| 13.5~13.6 | 20→7 | 80→93 |
| 13.6~19 | 7 | 93 |

**Preparation of reference solution**

Dissolve suitable amounts of loganin and morroniside reference substances in 80% methanol to obtain 50 µg/ml of each.

**Preparation of test solution**

Weigh about 0.15 g of the product, place in a stoppered conical flask, add 20 ml methanol, weigh, ultrasonicate (500 W, 40 kHz) for 30 minutes, cool, reweigh, replenish lost solvent with methanol, mix well, and filter.

**Determination method:**

Inject 1 µl each of the reference and test solutions into the HPLC system and record chromatograms. Each 1 g of product should contain 16.0 mg – 31.0 mg in total of loganin (C₁₇H₂₆O₁₁) and morroniside (C₁₇H₂₆O₁₀).

**[Specification]**

Each 1 g of formula granules is equivalent to 1.2 g of decoction pieces.

**[Storage]**

Sealed.

1. Licorice Formula Granules (XLS-09-TP-10242/R03)
2. **Purpose**

To establish the quality standard for *Glycyrrhizae Radix et Rhizoma* (Licorice) formula granule semi-finished product (National Standard), ensuring product quality and consistency.

1. **Applicable Scope**

Applicable to the quality inspection of the *Glycyrrhizae Radix et Rhizoma* (Licorice) formula granule semi-finished product (National Standard).

1. **Responsibility**

This standard shall be drafted, reviewed, and approved by the Quality Management Department. Quality inspection personnel must strictly follow this standard.

1. **Content**
   1. Basic Information
      1. Material Codes

Extract Code: H25015401

Granule Code: H35015401

- - 1. Reference Standards Chinese Pharmacopoeia (current edition); YBZ-PFKL-2021049
  1. Sampling Method

According to Sampling Operation Procedure *XLS-QA-OP-0003*.

- 1. **Standard Content**
     1. **Extract**

**[Description]** This product is a yellow to brownish-yellow powder with a slightly characteristic odor and a sweet, distinctive taste.

**[Identification]** Weigh 0.2 g of the sample and pulverize finely. Add 20 mL of water to dissolve, and extract twice with 20 mL of water-saturated n-butanol each time. Combine the n-butanol extracts, evaporate to dryness, and dissolve the residue in 5 mL of methanol to obtain the test solution.

Separately, weigh 0.5 g of *Glycyrrhizae Radix et Rhizoma* reference medicinal material, add 50 mL of water, boil for 30 minutes, filter, and concentrate the filtrate to 20 mL. Prepare the reference medicinal material solution in the same way.

Additionally, prepare a reference solution of ammonium glycyrrhizinate by dissolving an appropriate amount in methanol to obtain a solution containing 2 mg/mL.

Following the *Thin-layer Chromatography (TLC) Operating Procedure*, apply 2 μL each of the above three solutions on the same silica gel G plate (pretreated with 1% sodium hydroxide solution). Develop with a solvent system of ethyl acetate–formic acid–glacial acetic acid–water (15:1:1:2). Remove the plate, air dry, spray with 10% sulfuric acid in ethanol, heat at 105 °C until spots are clearly visible, and examine under UV light (365 nm).

In the chromatogram of the test solution, fluorescent spots of the same color appear at positions corresponding to those in the chromatograms of the reference medicinal material and the reference substance.

**[Characteristic Chromatogram]**

Determined according to the *High Performance Liquid Chromatography (HPLC) Method*.

**Chromatographic Conditions and System Suitability Test:**

Column: octadecylsilyl-bonded silica gel (C18), 100 mm × 2.1 mm, 2.2 µm

Mobile phase:

A: acetonitrile

B: 0.1% phosphoric acid solution

Gradient elution as specified in the following table

Flow rate: 0.3 mL/min

Column temperature: 30 °C

Detection wavelength: 237 nm

Theoretical plate number: not less than 5000, calculated with the glycyrrhizic acid peak.

| Time (min) | Mobile phase A (%) | Mobile phase B (%) |
| --- | --- | --- |
| 0～1 | 5→27 | 95→73 |
| 1～2 | 27 | 73 |
| 2～10 | 27→46 | 73→54 |
| 10～16 | 46→64 | 54→36 |
| 16～24 | 64→95 | 36→5 |
| 24～25 | 95 | 5 |

**Preparation of Reference Solution** Weigh 0.2 g of *Glycyrrhizae Radix et Rhizoma* (Licorice) reference medicinal material, place it in a stoppered conical flask, and add 100 mL of 70% ethanol. Seal the flask and ultrasonicate (power 250 W, frequency 40 kHz) for 30 minutes. Allow to cool, shake well, and filter. Collect the successive filtrate as the reference medicinal material solution.

Separately, accurately weigh appropriate amounts of liquiritin reference substance, isoliquiritigenin reference substance, and ammonium glycyrrhizinate reference substance. Dissolve them in methanol to prepare a reference substance mixed solution containing 0.1 mg/mL of liquiritin, 0.1 mg/mL of isoliquiritigenin, and 0.2 mg/mL of glycyrrhizic acid (calculated as glycyrrhizic acid = weight of ammonium glycyrrhizinate / 1.0207).

**Preparation of Test Solution** Prepare the test solution according to the method described under “Assay” (*Content Determination*).

**Determination Method** Accurately inject 1 μL each of the reference solution and test solution into the liquid chromatograph and determine under the specified chromatographic conditions.

In the chromatogram of the test solution, 12 characteristic peaks should be observed, corresponding in retention time to the 12 characteristic peaks in the chromatogram of the reference medicinal material solution. Among them, Peak 3, Peak 6, and Peak 10 should correspond to the retention times of liquiritin, isoliquiritigenin, and glycyrrhizic acid reference peaks, respectively.The peak corresponding to liquiritin is designated as S₁ peak. The relative retention times (RRT) of Peak 1 and Peak 2 with respect to the S₁ peak should be within ±10% of the specified values: peak 1: 0.83, peak 2: 0.98. The peak corresponding to isoliquiritigenin is designated as S₂ peak. The RRTs of Peak 4, Peak 5, and Peak 7 with respect to the S₂ peak should be within ±10% of the specified values: peak 4: 0.82, peak 5: 0.87, peak 7: 1.09. The peak corresponding to glycyrrhizic acid is designated as S₃ peak. The RRTs of Peak 8 to Peak 12 with respect to the S₃ peak should be within ±10% of the specified values: peak 8: 0.80, peak 9: 0.92, peak 11: 1.09, peak 12: 1.26.


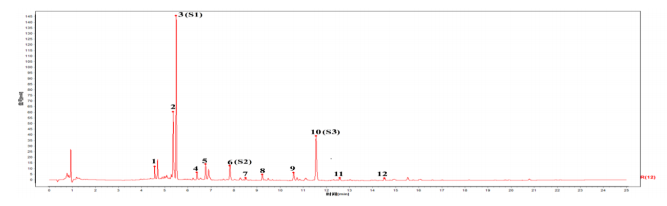


**Characteristic chromatogram peaks**

Peak 2: Liquiritin apioside, Peak 3 (S₁): Liquiritin, Peak 5: Isoliquiritin apioside, Peak 6 (S₂): Isoliquiritigenin, Peak 10 (S₃): Glycyrrhizic acid.

Column: Acclaim RSLC 120 C18 (2.1 mm × 100 mm, 2.2 μm)

**[Inspection]**

**Moisture:** Determine according to Method II described in the Moisture Determination Procedure. The moisture content shall not exceed 6.5%.

**Solubility:** Weigh 10 g of the sample, add 200 mL of hot water, stir for 5 minutes, and observe immediately. The sample should be completely dissolved or appear slightly turbid, with no presence of charred residues or foreign matter.

**Heavy Metals and Harmful Elements:** Determine according to the Operating Procedure for the Determination of Lead, Cadmium, Arsenic, Mercury, and Copper. The limits are as follows: Lead (Pb): ≤ 5 mg/kg, , Cadmium (Cd): ≤ 1 mg/kg, , Arsenic (As): ≤ 2 mg/kg, , Mercury (Hg): ≤ 0.2 mg/kg, Copper (Cu): ≤ 20 mg/kg.

**Organochlorine Pesticide Residues:** Determine according to Method I in the Operating Procedure for the Determination of Organochlorine and Other Pesticide Residues. The content of pentachloronitrobenzene shall not exceed 0.1 mg/kg.

**Extractives:** Determine according to the Procedure for Determination of Extractives, using the hot extraction method with ethanol as the solvent. The extractive content shall not be less than 35.2%.

**Content Determination:** Determine by High-Performance Liquid Chromatography (HPLC) according to the Operating Procedure for HPLC.

**Chromatographic Conditions and System Suitability Test** Use octadecylsilyl-bonded silica gel as the packing material (column: 100 mm × 2.1 mm, 2.2 μm). Use acetonitrile as mobile phase A and 0.05% phosphoric acid solution as mobile phase B, following the prescribed gradient elution program. Set the flow rate at 0.4 mL/min and the detection wavelength at 237 nm. Theoretical plate number, calculated with reference to the liquiritin peak, shall not be less than 5000.

| Time (min) | Mobile phase A (%) | Mobile phase B (%) |
| --- | --- | --- |
| 0~2 | 19 | 81 |
| 2~12.5 | 19→50 | 81→50 |
| 12.5~13 | 50→100 | 50→0 |
| 13~15 | 100→19 | 0→81 |

**Preparation of Reference Solution**

Accurately weigh appropriate amounts of liquiritin reference standard and ammonium glycyrrhizinate reference standard.Dissolve in 70% ethanol to prepare a solution containing 60 μg of liquiritin and 0.1 mg of ammonium glycyrrhizinate per 1 mL, respectively.(Note: The content of glycyrrhizic acid is calculated as glycyrrhizic acid ammonium weight / 1.0207.)

**Preparation of Test Solution**

Weigh an appropriate amount of the sample, grind finely, and accurately weigh about 0.1 g. Place it in a stoppered conical flask, add 50 mL of 70% ethanol, seal tightly, and weigh. Sonicate for 30 minutes (power: 250 W, frequency: 40 kHz), allow to cool, reweigh, and add 70% ethanol to compensate for any weight loss. Shake well, filter, and use the successive filtrate as the test solution.

**Determination**

Accurately inject 1 μL each of the reference solution and the test solution into the HPLC system, and determine. Each 1 g of the product shall contain liquiritin (C₂₁H₂₂O₉) in the range of 15.0–58.3 mg and glycyrrhizic acid (C₄₂H₆₂O₁₆) in the range of 29.0–133.3 mg.

**[Microbial Limit Test]**

Conduct the test according to the Operating Procedure for Microbiological Examination.

Total aerobic bacterial count: ≤ 2.0 × 10³ CFU/g (maximum acceptable limit: 2000 CFU/g). Total combined yeast and mold count: ≤ 2.0 × 10² CFU/g (maximum acceptable limit: 200 CFU/g). Escherichia coli: Not detectable.

- - 1. **Granules**

**Description:** This product consists of yellow to brownish-yellow granules with a slightly characteristic odor and sweet, distinctive taste.

**Inspection**

**Particle size:** Determine according to the Particle Size Determination Procedure; the proportion of fine particles shall not exceed 6.0%.

**Moisture:** Determine by Method II of the Moisture Determination Procedure; moisture content shall not exceed 6.5%.

**Solubility:** Weigh 10 g of the sample, add 200 mL of hot water, stir for 5 minutes, and observe immediately. The sample should completely dissolve or appear slightly turbid, with no charred residues or visible impurities.

1. Oyster Formula Granule (XLS-09-TP-15863/R00)

**1. Purpose**

To establish the quality standard for Oyster Formula Granule.

**2. Scope of Application**

This standard applies to the quality inspection of Oyster Formula Granule.

**3. Responsibility**

The Quality Management Department is responsible for drafting, reviewing, and approving this standard. Quality control personnel shall strictly implement this standard.

**4. Content**

**4.1 Basic Information**

**4.1.1 Material Codes**

Extract code: S25033301A1

Granule code: S35033301A1

**4.1.2 Reference Standards**

Based on the 2020 edition of the Chinese Pharmacopoeia and “Oyster (Omi-Oyster) Formula Granule SCYPBZ (PFKL)-2024034 (Trial)”.

**4.2 Sampling Method**

According to the Sampling Procedure (Document No. XLS-QA-OP-0003).

**4.3 Standard Specifications**

**4.3.1 Extract**

**Properties:** Off-white to yellowish-white powder; slight odor; mildly salty taste.

**Identification:**

Take 0.2 g sample, grind, add dilute HCl, observe effervescence.

Take 1 g sample, grind, add 5 ml dilute HCl, dissolve, filter, filtrate shows calcium salt reaction.

Take 0.5 g sample, grind, add 15 ml dilute HCl, effervescence occurs, ultrasonicate 30 minutes, adjust pH to 12 with NaOH solution, stand 10 minutes, centrifuge. Take precipitate into a 15 ml ampoule, add 10 ml 6.0 mol/L HCl, hydrolyze at 150℃ for 1 hour, cool, centrifuge, take supernatant, evaporate to dryness, dissolve residue in 1 ml methanol as test solution. Prepare reference solution from 2 g Omi-Oyster raw material similarly using dilute HCl. Perform Thin Layer Chromatography (TLC) according to procedure XLS-09-OP-2897, spotting 2 μl of each solution on a silica gel G plate, develop with n-butanol–glacial acetic acid–water–acetone–absolute ethanol–0.5% ninhydrin acetone solution (40:14:12:5:4:4), dry, and heat at 105℃ until clear spots appear. Test solution should show spots at the same position and color as reference.

**Tests:**

Heavy metals and harmful elements: Determined by Pb, Cd, As, Hg, Cu methods. Limits: Pb ≤ 5 mg/kg; Cd ≤ 1 mg/kg; As ≤ 2 mg/kg; Hg ≤ 0.2 mg/kg; Cu ≤ 20 mg/kg.

Moisture: Determined according to Moisture Determination Procedure (Method II), limit ≤ 6.5%.

**Assay:**

Take ~0.1 g sample, grind, place in conical flask, add 10 ml dilute HCl, dissolve with heat, cool, compensate weight with dilute HCl, shake, filter. Take 5 ml filtrate, add 20 ml water and 1 drop methyl red, titrate with 10% KOH until light yellow, add 5 ml more KOH, add small amount of calcium green indicator, titrate with 0.05 mol/L EDTA until yellow-green fluorescence disappears and solution turns orange. 1 ml EDTA = 5.004 mg CaCO₃. Content per gram: 150–640 mg CaCO₃.

**Microbial Limits:**

Total aerobic bacteria ≤ 10³ CFU/g (Max 2000 CFU/g); Molds and yeasts ≤ 10² CFU/g (Max 200 CFU/g); E. coli: Not detected; Salmonella: Not detected.

**4.3.2 Granules**

**Properties:** Off-white to yellowish-white granules; slight odor; mildly salty taste.

**Tests:**

Particle size: Determined according to Particle Size Determination Procedure, limit ≤ 6.0%.

Moisture: Determined according to Moisture Determination Procedure (Method II), limit ≤ 6.5%.

1. Calcined Magnetite Formula Granules (XLS-09-TP-15876/R00)

**1. Purpose:**

To establish the quality standard for the product of *Calcined Magnetite Formula Granules*to ensure product quality.

**2. Applicable Scope:**

Quality testing product of *Calcined Magnetite Formula Granules*.

**3. Responsibility:**

The Quality Management Department is responsible for drafting, reviewing, and approving this standard. Quality inspection personnel shall strictly implement it.

**4. Content:**

**4.1 Basic Information**

**4.1.1 Material Code**

Extract code: S25009021A1

Granule code: S35009021A1

**4.1.2 Standard Basis:**

*Chinese Pharmacopoeia* (2020 edition), “Calcined Magnetite Formula Granules SCYPBZ (PFKL)-2024008 (Trial)”.

**4.2 Sampling Method:**

Sampling according to SOP XLS-QA-OP-0003.

**4.3 Standard Content**

**4.3.1 Extract**

**Appearance:**

Light gray to gray powder; slightly odorous, tasteless.

**Identification:**

Take an appropriate amount of the sample, grind it finely, take 0.1 g, add 10 mL of hydrochloric acid, shake to dissolve, and allow to stand.

(1) Take the supernatant, add potassium ferrocyanide solution — a deep blue precipitate appears; it is insoluble in dilute hydrochloric acid. Centrifuge, add sodium hydroxide solution to the precipitate, and mix — a yellow-brown precipitate forms.

(2) Take the supernatant, add ammonium thiocyanate solution — a blood-red color appears.

**Tests:**

**Moisture:** Determined according to “Moisture Determination Procedure” (XLS-09-OP-2873), Method II; moisture should not exceed 6.5%.

**Assay:**

Accurately weigh about 0.5 g of the finely powdered sample into a conical flask. Add 15 mL of hydrochloric acid and 3 mL of 25% potassium fluoride solution. Cover with a watch glass and heat to gentle boiling. Add 6% stannous chloride solution [1] dropwise with constant shaking until completely decomposed (a white residue remains). Rinse the flask and watch glass with a small amount of water. While hot, add 6% stannous chloride solution until light yellow (if excessive, add potassium permanganate solution until light yellow). Add 100 mL water, 15 drops of 25% sodium tungstate solution [2], and titrate with 0.01667 mol/L potassium dichromate solution after adding 1% titanium trichloride solution [3] until the blue color just disappears. Immediately add 10 mL of sulfuric acid–phosphoric acid–water (2:3:5) and 20 drops of diphenylamine sulfonate indicator solution, and continue titration with potassium dichromate solution until a stable blue-violet color appears.

Each 1 mL of 0.01667 mol/L potassium dichromate solution equals 5.585 mg of Fe.

The content of Fe per 1 g of sample should be 5.0 mg – 94.1 mg.

**Notes:**

[1] 6% Stannous Chloride Solution: Dissolve 6 g of stannous chloride in 20 mL hydrochloric acid with heating, cool, and dilute to 100 mL with water.

[2] 25% Sodium Tungstate Solution: Dissolve 25 g sodium tungstate in 2 mL phosphoric acid, dilute to 100 mL with water.

[3] 1% Titanium Trichloride Solution: Take an appropriate amount of titanium trichloride solution, add 2 mL hydrochloric acid, dilute to 100 mL with water (final concentration 1%).

**Microbial Limit Tests:**

Conduct according to *Microbial Examination Procedure.*

Total aerobic bacteria: ≤ 10³ CFU/g (maximum acceptable 2000 CFU/g)

Total molds and yeasts: ≤ 10² CFU/g (maximum acceptable 200 CFU/g)

*Escherichia coli:* Not detectable

**4.3.2 Granules**

**Appearance:**

Light gray to gray granules; slightly odorous, tasteless.

**Tests:**

**Particle Size:** Determined according to “Particle Size Determination Procedure” (XLS-09-OP-2874); should not exceed 6.0%.

**Moisture:** Determined according to “Moisture Determination Procedure” (XLS-09-OP-2873), Method II; moisture should not exceed 6.5%.

1. Fossilia Ossia Mastodi (Longgu) Formula Granule (XLS-09-TP-17619/R00)

**1. Purpose**

To establish the quality standard for fossilia ossia mastodi (Longgu) Formula Granule.

**2. Scope of Application**

This standard applies to the quality inspection of fossilia ossia mastodi formula granule.

**3. Responsibility**

The Quality Management Department is responsible for drafting, reviewing, and approving this standard. Quality control personnel shall strictly implement the requirements of this standard.

**4. Content**

**4.1 Basic Information**

**4.1.1 Material Codes**

Extract code: C80029301A101

Granule code: C90CK9L01101A1100

**4.1.2 Reference Standards**

Based on the current edition of the Chinese Pharmacopoeia and “Fossilia Ossia Mastodi Formula Granule (Trial) SCYPBZ (PFKL)-2025024 (Trial)”.

**4.2 Sampling Method**

According to the Sampling Procedure (Document No. XLS-QA-OP-0003).

**4.3 Standard Specifications**

This product is made from fossilized bones of ancient mammals including three-toed horses, rhinoceroses, deer, cattle, elephants, or elephant incisors, processed according to decoction standards and key quality indicators to produce formula granules.

**[Manufacturing Method]**

Take 25,000 g of fossilia ossia mastodi decoction pieces, decoct with water, filter, and concentrate the filtrate to a clear paste (yield of dry extract: 1.5%–2.8%). Add appropriate excipients, dry (or dry and pulverize), mix with additional excipients as required, granulate, and produce 1,000 g of granules.

**[Properties]**

Light gray-white to yellowish-white granules; slight odor; bland taste.

**[Identification]**

Carbonate: Take 2 g of the sample, grind, add 10 ml dilute acid, observe the carbonate reaction.

Calcium salt: Take 2 g, grind, add 10 ml dilute acid, observe the calcium reaction.

Phosphate: Take 2 g, grind, add 10 ml dilute acid, observe the phosphate reaction.

**[Tests]**

Particle size: Tested according to Particle Size Determination Procedure. Limit: ≤ 15%.

Moisture: Tested according to Moisture Determination Procedure (Method II). Limit: ≤ 8.0%.

Fill weight variation (single-dose packaging): Take 10 sachets, remove packaging, precisely weigh each sachet content. Sachets exceeding the fill weight limit shall not exceed 2, and no sachet may exceed the limit by more than one-fold. Comply with Fill Weight Determination Procedure. Fill weight limits:

| Labeled amount | Fill weight variation limit |
| --- | --- |
| ≤ 1.0 g | ±10% |
| >1.0–1.5 g | ±8% |
| >1.5–6.0 g | ±7% |
| >6.0 g | ±5% |

Fill weight (multi-dose packaging): Take 3 containers, clean and dry, weigh empty and filled containers, calculate content per container. Average content must not be less than labeled amount; individual container content ≥ 97% of labeled amount. If one container fails, retest 3 more containers; all must comply.

**[Assay]**

Take 0.1 g sample, grind, place in conical flask, add 5 ml dilute HCl, ultrasonicate to dissolve, add 200 ml water and 1 drop methyl red, titrate with 10% KOH to yellow, add 2 ml tartaric acid solution (1→5) and 2 ml triethanolamine (3→10), shake, add 10 ml 10% KOH, add small amount of calcein indicator, titrate with 0.05 mol/L EDTA to blue color that persists 30 seconds. 1 ml EDTA = 5.004 mg CaCO₃.

Content per gram: 200–690 mg CaCO₃.

**[Microbial Limits]**

Total aerobic bacteria ≤ 10³ CFU/g (Max 2000 CFU/g); Molds and yeasts ≤ 10² CFU/g (Max 200 CFU/g); E. coli: Not detected; Salmonella: Not detected.

**[Specifications]**

1 g granules equivalent to 25 g decoction pieces.

**[Packaging]**

Aluminum-plastic composite film.

**[Storage]**

Sealed.

**[Shelf Life]**

Three years.

**4.3.1 Internal Control Standard (Provincial Standard Internal Control)**

| Parameter | Legal Standard | Internal Control Standard |
| --- | --- | --- |
| Content | CaCO₃: 200–690 mg | CaCO₃: 203–680 mg |
