# Supplementary material for: Poge heart-saving decoction meliorates heart failure by suppressing apoptosis and fibrosis via regulation of the PI3K/AKT pathway
Source: Front Pharmacol. 2026 Mar 25;17:1748420. doi: 10.3389/fphar.2026.1748420 (PMC13058608; doi:10.3389/fphar.2026.1748420)
Supplement: Supplementary file 4 [file Supplementaryfile2.docx]

Table B.1 The gradient elution conditions

| Time (min) | Flow Rate (mL/min) | %A | %B |
| --- | --- | --- | --- |
| 0 | 0.3 | 100 | 0 |
| 10 | 0.3 | 70 | 30 |
| 25 | 0.3 | 60 | 40 |
| 30 | 0.3 | 50 | 50 |
| 40 | 0.3 | 30 | 70 |
| 45 | 0.3 | 0 | 100 |
| 60 | 0.3 | 0 | 100 |
| 60.5 | 0.3 | 100 | 0 |
| 70 | 0.3 | 100 | 0 |

A: deionized water containing 0.1% formic acid. B: acetonitrile containing 0.1% formic acid

Table B.2 Antibodies and reagents information used in the study

| Antibodies and reagents | Manufacturers, city, countries | Cat.No. |
| --- | --- | --- |
| Rabbit polyclonal antibody to PI3K | Affinity, Jiangsu, China | AF6242 |
| Rabbit polyclonal antibody to AKT | CST, Massachusetts, USA | 4691 |
| Rabbit polyclonal antibody to Bax | Huabio, Hangzhou, China | ET1603-34 |
| Rabbit polyclonal antibody to Bcl-2 | Huabio, Hangzhou, China | ET1702-53 |
| Rabbit polyclonal antibody to p-PI3K | Affinity，Jiangsu, China | AF3241 |
| Rabbit polyclonal antibody to p-AKT | CST，Massachusetts, USA | 4060 |
| Rabbit polyclonal antibody to Collagen I | Affinity，Jiangsu, China | AF7001 |
| Rabbit polyclonal antibody to CollagenШ | Affinity，Jiangsu, China | AF5457 |
| Rabbit polyclonal antibody to GAPDH | Huabio, Hangzhou, China | R1210-1 |
| HRP-labeled Goat Anti-Rabbit IgG (H+L) | Beyotime, Shanghai, China | A0208 |
| Mouse NT-proBNP assay kit | Mlbio, Shanghai, China | ml324452 |
| Isoprenaline | MedChemExpress, New Jersey, USA | HY-B0468 |
| Deslanoside | MedChemExpress, New Jersey, USA | HY-A0154 |
| Hematoxylin and Eosin Staining Kit | Beyotime, Shanghai, China | C0105S |

Table B.3 Dose conversion for mice

|  | PHSD | Deslanoside |
| --- | --- | --- |
| Standard human weight | 70kg | |
| Clinical dose | 360 g/day^*^ | 0.4mg/day |
| Human equivalent dose^&^ | 5.14g/kg | 0.006mg/kg |
| Mice dose^#^ | 46.8g/kg | 0.052mg/kg |
|  |  |  |

* 360g=60g(Aconite Tuber) + 60g(Ginger Rhizome) + 60g(Red Ginseng) + 60g(Cornus) + 30g(licorice) + 30g(Ossa Draconis) + 30g(Oyster) + 30g(Magnetitum)

& Human equivalent dose = Clinical dose / standard human weight

# mice dose=human equivalent dose x 9.1. The mice dose was calculated based on body surface area normalization.
